# Tailored psychological intervention for anxiety or depression in COPD (TANDEM): a randomised controlled trial

**DOI:** 10.1183/13993003.00432-2023

**Published:** 2023-11-02

**Authors:** Stephanie J.C. Taylor, Ratna Sohanpal, Liz Steed, Karen Marshall, Claire Chan, Nahel Yaziji, Amy C. Barradell, Paulino Font-Gilabert, Andrew Healey, Richard Hooper, Moira J. Kelly, Kristie-Marie Mammoliti, Stefan Priebe, Arvind Rajasekaran, C. Michael Roberts, Vickie Rowland, Sally J. Singh, Melanie Smuk, Martin Underwood, Sarah Waseem, Patrick White, Vari Wileman, Hilary Pinnock

**Affiliations:** 1 Wolfson Institute of Population Health, Queen Mary University of London, London, UK; 2 Chest Clinic, RVI Hospital, Newcastle upon Tyne NHS Foundation Trust, Newcastle upon Tyne, UK; 3 Health Service and Population Research Department, Institute of Psychiatry, Psychology and Neuroscience, King's College London, London, UK; 4 NIHR Leicester Biomedical Research Centre – Respiratory, Glenfield Hospital, University Hospitals of Leicester NHS Trust, Leicester, UK; 5 WHO Collaborating Centre on Global Women's Health, Institute of Metabolism and Systems Research, University of Birmingham, Birmingham, UK; 6 Department of Respiratory Medicine, Sandwell and West Birmingham Hospitals NHS Trust, Birmingham, UK; 7 Centre for Digital Transformation of Health, University of Melbourne, Melbourne, Australia; 8 Department of Population Health, School of Life Course and Population Sciences, King's College London, London, UK; 9 Department of Respiratory Sciences, Department of Health Sciences, University of Leicester, Leicester, UK; 10 Blizard Institute, Queen Mary University of London, London, UK; 11 Warwick Clinical Trials Unit, University of Warwick, Coventry, UK; 12 University Hospitals of Coventry and Warwickshire, Coventry, UK; 13 Women's Health Division, University College Hospital, London, UK; 14 Health Psychology, School of Mental Health and Psychological Sciences, Institute of Psychiatry, Psychology and Neuroscience, King's College London, London, UK; 15 Allergy and Respiratory Research Group, Usher Institute, The University of Edinburgh, Edinburgh, UK; 16 Joint first authors

## Abstract

**Background:**

The TANDEM multicentre, pragmatic, randomised controlled trial evaluated whether a tailored psychological intervention based on a cognitive behavioural approach for people with COPD and symptoms of anxiety and/or depression improved anxiety or depression compared with usual care (control).

**Methods:**

People with COPD and moderate to very severe airways obstruction and Hospital Anxiety and Depression Scale subscale scores indicating mild to moderate anxiety (HADS-A) and/or depression (HADS-D) were randomised 1.25:1 (242 intervention and 181 control). Respiratory health professionals delivered the intervention face-to-face over 6–8 weeks. Co-primary outcomes were HADS-A and HADS-D measured 6 months post-randomisation. Secondary outcomes at 6 and 12 months included: HADS-A and HADS-D (12 months), Beck Depression Inventory II, Beck Anxiety Inventory, St George's Respiratory Questionnaire, social engagement, the EuroQol instrument five-level version (EQ-5D-5L), smoking status, completion of pulmonary rehabilitation, and health and social care resource use.

**Results:**

The intervention did not improve anxiety (HADS-A mean difference −0.60, 95% CI −1.40–0.21) or depression (HADS-D mean difference −0.66, 95% CI −1.39–0.07) at 6 months. The intervention did not improve any secondary outcomes at either time-point, nor did it influence completion of pulmonary rehabilitation or healthcare resource use. Deaths in the intervention arm (13/242; 5%) exceeded those in the control arm (3/181; 2%), but none were associated with the intervention. Health economic analysis found the intervention highly unlikely to be cost-effective.

**Conclusion:**

This trial has shown, beyond reasonable doubt, that this cognitive behavioural intervention delivered by trained and supervised respiratory health professionals does not improve psychological comorbidity in people with advanced COPD and depression or anxiety.

## Tweetable abstract

**The TANDEM trial reports no benefit from a cognitive behavioural approach intervention focused particularly on breathlessness in people with advanced COPD and anxiety/depression. More research is needed for these patients with high levels of unmet need.** https://bit.ly/3QDCB8v

## Introduction

COPD is a complex, multisystem condition associated with comorbidities that adversely affect quality of life and survival [1]. Anxiety and depression are very common comorbidities with a prevalence of 30–40% and higher rates with more severe COPD [2, 3]. Anxiety and/or depression reduce people's ability to manage their COPD effectively, reduce physical activity, capacity and capability, and increase susceptibility to exacerbations, hospital admission and readmission [3, 4]. A 2019 Cochrane review (13 studies, n=1500) concluded that psychological interventions, including cognitive behavioural therapy, may improve depression in people with COPD, but that current evidence is limited by small studies at high risk of bias [5].

Pulmonary rehabilitation, a multidisciplinary exercise and education intervention designed to reduce the symptom burden associated with COPD-induced deconditioning, improves health-related outcomes including functional exercise capacity, quality of life and emotional wellbeing, and reduces breathlessness and symptoms of anxiety and depression [6, 7]. National and international COPD guidelines recommend referring patients who are functionally disabled by breathlessness for pulmonary rehabilitation. However, over one-third of people referred to pulmonary rehabilitation in Britain do not attend and only two-thirds who attend complete the course [8]. People from more deprived circumstances, those with worse disease and those who are depressed are less likely to complete pulmonary rehabilitation [8, 9].

A systematic review of complex interventions concluded that psychological interventions combined with exercise training resulted in greater improvements in symptoms of anxiety and depression in COPD compared with cognitive behavioural therapy alone (although not all the studies were limited to people with anxiety and depression at baseline) [10]. Thus psychological interventions, particularly cognitive behavioural approaches, and pulmonary rehabilitation both have potential to improve psychological wellbeing in people with COPD and comorbid anxiety or depression, raising the possibility of synergistic effects [5, 6]. In addition, improving mood or anxiety might support people to attend pulmonary rehabilitation and complete a course [9]. We designed a tailored, psychological cognitive behavioural approach intervention (referred to as TANDEM (Tailored intervention for ANxiety and DEpression Management)), which preceded attending an existing, routine pulmonary rehabilitation course, with the aim of reducing symptoms of anxiety and depression and promoting the uptake and completion of pulmonary rehabilitation in people with COPD. Here we report our evaluation of the effectiveness and cost-effectiveness of TANDEM in people with symptoms of mild to moderate anxiety and/or depression and moderate to very severe COPD.

## Methods

The TANDEM trial was a multicentre, parallel-group, individually randomised, pragmatic, controlled trial, comparing a tailored, cognitive behavioural approach intervention with usual care for people with COPD and symptoms of mild to moderate anxiety and/or depression. Detailed methods are provided in the trial protocol and statistical analysis plans published elsewhere [11, 12]. All protocol changes underwent ethics and governance approvals and are described in supplementary table S1. The trial was approved by the London – Queen Square Research Ethics Committee (17/LO/0095). All participants provided written, informed consent. The trial was registered on 20 March 2017 with the ISRCTN registry (ISRCTN59537391).

### Participants

Between 2017 and 2020, participants were screened and recruited from primary and secondary care across 17 geographically dispersed NHS organisations (NHS Trusts) in England. Eligible participants had a spirometry-confirmed diagnosis of COPD with moderate to very severe airflow limitation and were eligible for referral to their local pulmonary rehabilitation service [13]. On screening, eligible participants had a Hospital Anxiety and Depression Scale (HADS) score suggestive of mild to moderate anxiety, depression or both (*i.e.* HADS depression (HADS-D) or anxiety (HADS-A) subscale scores in the range ≥8 to ≤15) [14]. Those with scores suggesting severe anxiety/depression were ineligible and were referred to their general practitioners for more intensive mental health support. Participants were sufficiently fluent in spoken English to be able to receive the intervention but did not have to be able to read or write English. People who had received a psychological intervention for anxiety or depression within the preceding 6 months were excluded, but those taking psychotropic medication were eligible.

Following baseline data collection participants were randomised to intervention or usual care (control) using a centralised, online service and stratified by NHS Trust. Within each NHS Trust minimisation balanced allocations according to baseline HADS subscale scores, breathlessness determined by the modified Medical Research Council breathlessness scale (mMRC) and smoking status [12]. Study personnel involved in the collection and processing of outcome data were masked to participants’ allocation and any accidental unmasking was recorded. Participants’ healthcare providers were unaware of their allocation.

### Procedures

The TANDEM intervention was designed to take place in the hiatus between referral for assessment for pulmonary rehabilitation and commencement of a course (in 2017 median wait ∼11 weeks in England). Although TANDEM was intended to complement pulmonary rehabilitation it was a stand-alone intervention so that participants who did not attend pulmonary rehabilitation could still benefit. Full details of the intervention have been published previously [15]. In summary, we recruited and trained respiratory professionals with experience of working with people with COPD as TANDEM facilitators to deliver the intervention (see supplementary box S1).

Throughout intervention delivery the facilitators received telephone supervision from an experienced cognitive behavioural therapist. TANDEM was a tailored, manualised intervention based on a cognitive behavioural approach focusing on the inter-related physical symptoms, thoughts, feelings and behaviours associated with living with COPD, with a particular focus on breathlessness. Self-management support, delivered as required, incorporated material from the “Self-management Programme of Activity, Coping and Education for COPD” manual and the British Lung Foundation charity [16]. The intervention was delivered over 6–8 weeks and is summarised in table 1. On completion of the face-to-face intervention, facilitators offered brief, weekly telephone support for up to 2 weeks after completing pulmonary rehabilitation. The facilitators’ manual is available from the corresponding author. Our approach to promoting and measuring the fidelity of intervention delivery, and the competence of the TANDEM facilitators, has been published previously [17]. Participants randomised to the control arm received usual care including routine referral to pulmonary rehabilitation.

**TABLE 1:** Summary of TANDEM face-to-face intervention content

**Weekly session^#,^** ^¶^	**Topics covered^#^**	**Content**
**Week 1**	Introduction, setting expectations Topic 1: What is COPD? Topic 2: Taking control of COPD Topic 3: The patient experience of breathlessness	Eliciting the patient’s understanding of COPD Identifying and working with illness and treatment beliefs and acceptance Teaching basic breathing control Suggestions for home practice^+^
**Week 2**	Feedback from home practice^+^ Topic 4: Introducing mood and COPD	Conducting a formulation and presentation of a cognitive behavioural approach
**Weeks 3–6**	Feedback from home practice Topic 5: Managing anxiety and COPD Topic 6: Managing depression and COPD Topic 7: Applying the cognitive behavioural approach to other problems (optional)	Two to four sessions, tailored to participant needs – conduct cognitive behavioural work on anxiety and/or depression dependent on individual need One session available to discuss other problems, if needed
**Weeks 5–7**	Feedback from home practice Topic 8: Living with COPD day to day	Self-management approaches to COPD Learning to problem solve and set goals
**Weeks 6–8**	Feedback from home practice Topic 9: Preparing for pulmonary rehabilitation	Expectations of pulmonary rehabilitation, addressing worries and concerns

^#^: weeks are indicative only, topics could have been covered in adjacent weeks as required by individual participants, weeks/sessions could be repeated if participants had their intervention interrupted by illness or hospitalisation. ^¶^: weekly face-to-face sessions last 40–60 min in participants’ own homes or, if participants prefer, in a convenient local healthcare setting. Delivery was by telephone during the coronavirus disease 2019 pandemic restrictions. With participant consent, all sessions were recorded on an encrypted digital audio recorder to assess fidelity. ^+^: between sessions home practice exercises were suggested in agreement with participants.

Baseline data were collected face-to-face as supervised, self-complete questionnaires. Follow-up data at 6 and 12 months could also be collected by post or phone if participants preferred.

### Outcomes

The co-primary outcomes were symptoms of anxiety and depression 6 months post-randomisation, measured using the HADS anxiety (HADS-A) and depression (HADS-D) scores [14]. Secondary outcomes were: HADS-A and HADS-D at 12 months, and at both 6 and 12 months: Beck Depression Inventory II (BDI II) and Beck Anxiety Inventory (BAI) [18, 19], health-related quality of life using the St George's Respiratory Questionnaire (SGRQ) [20], social engagement and support using the five-question Social Integration and Support subscale of the Health Education Impact Questionnaire [21], social activity using an adapted version of the UK Time Use Survey [22], the Brief Illness Perception Questionnaire [23], and the EuroQol instrument five-level version (EQ-5D-5L) [24]. Further details on questionnaire outcomes assessed can be found in supplementary table S2.

Data were also collected on uptake and completion of pulmonary rehabilitation (defined as attending 75% of scheduled sessions) in the 12 months following recruitment, and tobacco consumption or e-cigarette use at 6 and 12 months. We collected medication use data at baseline and 12 months and health and social care resource use over 12 months following recruitment.

### Statistical analysis

The sample size of 430 participants (240 in the intervention arm and in the 190 control arm) was calculated to achieve 90% power to detect a difference of 1.7 points on the HADS-A subscale and 1.5 points on the HADS-D subscale at the 2.5% significance level, allowing for 20% dropout and assuming standard deviations of 4.2 for anxiety and 3.6 for depression, a therapist intraclass correlation coefficient of 0.01 and mean cluster size of 24 at randomisation [25]. These effect sizes equate to a standardised mean difference of ∼0.4 and are in line with the minimum clinically important difference for HADS in COPD [26]. Since only the intervention arm was clustered (by facilitator), an allocation ratio of 1.25:1 was adopted to reduce the sample size [27].

The primary analysis was by intention to treat assuming outcomes were missing at random. We explored the robustness of this assumption through sensitivity analyses. A secondary analysis estimated the complier average causal effect (CACE) (for more details, see the statistical analysis plan in the supplementary material) [12]. All outcomes other than smoking were analysed using a mixed linear regression model with adjustment for fixed effects of baseline HADS-A and HADS-D, breathlessness, smoking status, NHS Trust and (except for HADS scores) the measurement of that outcome at baseline. Analyses allowed for clustering in the intervention arm by adjusting for a random effect of facilitator. Individual participants in the control arm were treated as clusters of size one. The mixed model allowed for heteroscedasticity to distinguish between clusters defined by a facilitator and clusters of one individual [28]. A Satterthwaite correction was applied to correct for the relatively small number of clusters [28].

We intended the analyses of smoking status at 6 and 12 months to work in a similar way using a mixed logistic regression model. The statistical analysis plan prespecified a strategy of successive steps for simplifying analyses in the event that there were problems with model convergence. The final analyses of smoking status used separate models for 6- and 12-month smoking outcome, and did not adjust for any covariates, but did include a random effect of facilitator. The final version of the statistical analysis plan, together with details of any changes to the first version [12], and full details of all sensitivity analyses including those for the coronavirus disease 2019 (COVID-19) pandemic are provided in the supplementary material.

During the trial, an anomaly in the allocation ratio produced by the online randomisation system became apparent: over the first 69 randomisations the observed allocation ratio was ∼1.25:1 as expected, but over the next 70 randomisations the ratio was ∼5:1. This triggered a corrective action and prevention plan overseen by the sponsor and with the approval of the data monitoring and ethics committee, that included migrating the randomisation system to a new platform. For the remaining randomisations an allocation ratio of 1:1 was specified to return the overall allocation ratio to a figure closer to 1.25:1 and the observed allocation ratio over this third randomisation period was close to 1:1. Full details including baseline characteristics of participants recruited across these time periods and additional sensitivity analyses are provided in the supplementary material.

We also conducted a health economic evaluation adopting a “cost-utility” framework, with the incremental resource impact of TANDEM over usual care quantified from an NHS/Personal Social Services perspective and patient outcomes quantified as incremental quality-adjusted life years (QALYs) gained (full details in supplementary box S3).

The impact of the COVID-19 pandemic on the study is described in supplementary box S4.

## Results

### Participants

**FIGURE 1:**
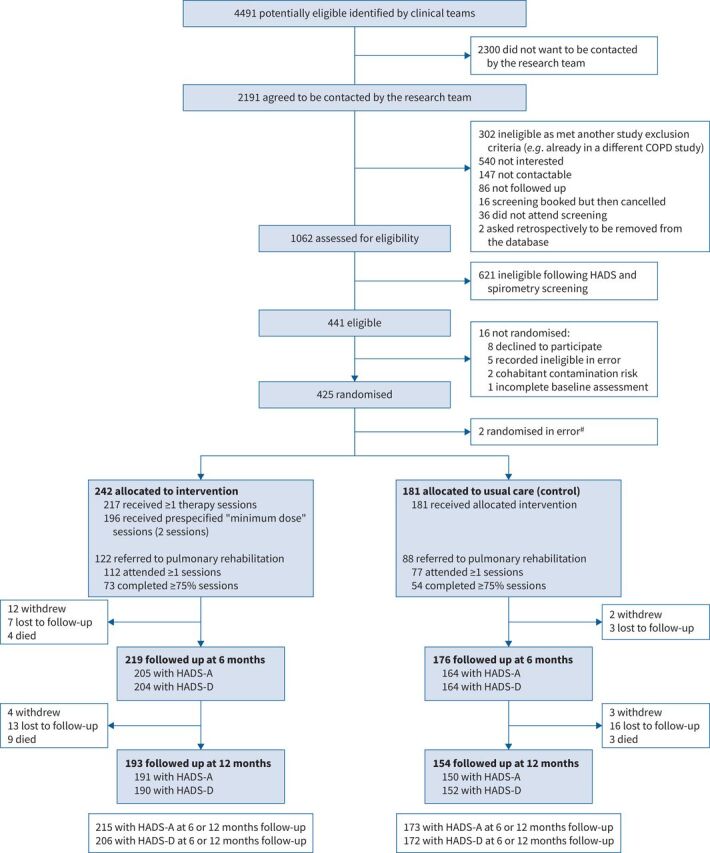
Trial profile. ^#^: two participants were randomised (both to the intervention) in error as they did not meet the eligibility criteria. They were picked up during the ongoing data cleaning process for data monitoring and ethics committee reports and thus were found in an unbiased way. These two participants were excluded from all analyses.

In total, 4491 potential participants were approached in person by clinicians as they attended primary, community or secondary care clinics, during admission for an acute exacerbation of COPD, or by invitation letter from their general practitioner (figure 1). Of those approached, 2191 (49%) agreed to be contacted by the research team, of whom 1062 (48%) were potentially eligible and proceeded to formal screening for the study. Of those screened, 441 (42%) met the trial eligibility criteria and 425 (96%) were randomised. Two randomised participants were subsequently noted to be ineligible and excluded from analyses. Of those remaining, 242 were randomised to intervention and 181 to usual care (control); at 6 months, 219 (90%) intervention and 176 (97%) control participants were followed up. Co-primary outcome measures were available for 205/204 (HADS-A/HADS-D, 85%/84%) intervention arm participants and 164 (both HADS subscales, 91%) control arm participants. At 12 months, follow-up data were available on 193 (80%) intervention and 154 (85%) control participants. Outcome assessors remained masked to participant's allocation arm in 408/423 cases (96%). More participants withdrew from the intervention arm (16 (7%)) compared with the control arm (5 (3%)). There were more deaths in the intervention arm compared with the control arm (13 (5%) *versus* 3 (2%)); no deaths were unexpected or associated with the intervention and most deaths occurred in the second 6 months of follow-up.

Median (interquartile range) age at baseline was 69 (62–75) years, 213 (50%) were male and 176 (42%) lived alone (table 2). Most participants reported significant breathlessness, 80% (340) being at mMRC grade 2 or worse, with 18% (78) too breathless to leave the house (grade 4), 41% (175) had previously attended pulmonary rehabilitation, 90% (381) reported comorbidities and 30% (128) were current smokers. Overall, intervention and control participants had similar baseline characteristics (table 2).

**TABLE 2:** Baseline characteristics and questionnaire responses by treatment allocation

	**Treatment arm**		**Overall**	**n (data available):** **all (intervention/usual care)**
	**Intervention (n=242)**	**Usual care (n=181)**		
**Age (years)**	68 (61–76)	69 (63–74)	69 (62–75)	411 (237/174)
**Gender**				422 (242/180)
Male	130 (53.7)	83 (46.1)	213 (50.5)	
Female	112 (46.3)	97 (53.9)	209 (49.5)	
**Living circumstances**				419 (239/180)
Lives alone	108 (45.2)	68 (37.8)	176 (42.0)	
Lives with spouse or partner	83 (34.7)	72 (40.0)	155 (37.0)	
Lives with adult family member	33 (13.8)	22 (12.2)	55 (13.1)	
**In paid employment/working**	26 (10.8)	14 (7.8)	40 (9.5)	420 (241/179)
If working, hours per week in paid employment/working	27 (15–37)	36 (35–38)	35 (16–37)	39 (26/13)
**Formal education, age completed full-time education**				416 (238/178)
Had formal education	238 (98.3)	178 (98.9)	416 (98.6)	422 (242/180)
≤12 years	4 (1.7)	5 (2.8)	9 (2.2)	
13–16 years	190 (79.8)	139 (78.1)	329 (79.1)	
17–18 years	27 (11.3)	13 (7.3)	40 (9.6)	
>18 years	17 (7.1)	21 (11.8)	38 (9.1)	
**COPD**				
Age first diagnosed with COPD (years)	60 (53–68)	60 (53–67)	60 (53–67)	401 (234/167)
Recent (previous 6 months) hospitalisation for COPD exacerbation	58 (24.0)	50 (27.6)	108 (25.5)	
On home oxygen	18 (7.5)	13 (7.3)	31 (7.4)	419 (240/179)
Attended pulmonary rehabilitation previously	99 (40.9)	76 (42.2)	175 (41.5)	422 (242/180)
**Other long-term health problems^#^**				423 (242/181)
Heart disease	36 (14.9)	25 (13.8)	61 (14.4)	
Diabetes	39 (16.1)	25 (13.8)	64 (15.1)	
Arthritis	91 (37.6)	70 (38.7)	161 (38.1)	
High blood pressure	90 (37.2)	72 (39.8)	162 (38.3)	
Asthma	60 (24.8)	49 (27.1)	109 (25.8)	
Epilepsy	7 (2.9)	2 (1.1)	9 (2.1)	
Other	120 (49.6)	91 (50.3)	211 (49.9)	
None	26 (10.7)	16 (8.8)	42 (9.9)	
**Smoking status**				423 (242/181)
Current smoker	74 (30.6)	54 (29.8)	128 (30.3)	
Ex-smoker	162 (66.9)	124 (68.5)	286 (67.6)	
Never-smoker	6 (2.5)	3 (1.7)	9 (2.1)	
Current smoker: pack-years	14.5 (7.2–30.0)	25.0 (5.0–46.2)	17.1 (6.0–34.7)	107 (62/45)
Current vaper (including e-cigarettes)	7 (14.0)	10 (21.3)	17 (17.5)	97 (50/47)
**mMRC grade**				443 (242/181)
0: Not troubled by breathlessness except on strenuous exercise	3 (1.2)	0 (0.0)	3 (0.7)	
1: Short of breath when hurrying on the level or walking up a slight hill	42 (17.4)	38 (21.0)	80 (18.9)	
2: Walks slower than other people of the same age on the level	79 (32.6)	57 (31.5)	136 (32.2)	
3: Stops for breath after walking about 100 yards (91 m) or after a few minutes on the level	75 (31.0)	51 (28.2)	126 (29.8)	
4: Too breathless to leave the house or breathless when dressing or undressing	43 (17.8)	35 (19.3)	78 (18.4)	
**HADS^¶^**				422 (242/180)
HADS-A total score	9.7±3.1	9.9±3.3	9.8±3.2	423 (242/181)
HADS-D total score	9.2±3.1	9.1±3.1	9.1±3.1	423 (242/181)
**BDI II and BAI**				
BDI II total score	20.2±8.8	20.7±10.2	20.4±9.4	402 (234/168)
BAI total score	16.6±10.3	16.6±10.2	16.6±10.2	389 (223/166)
**SGRQ**				
Overall score	59.6±15.1	58.6±15.4	59.2±15.2	418 (240/178)
Symptoms score	63.8±20.7	62.4±23.2	63.2±21.8	422 (242/180)
Activity score	78.6±18.2	77.6±15.9	78.2±17.2	419 (240/179)
Impact score	47.4±17.0	46.7±18.1	47.1±17.4	419 (240/179)
**B-IPQ**				
Consequences score	6.4±2.1	6.6±2.2	6.5±2.2	418 (240/178)
Timeline score	9.5±1.3	9.4±1.5	9.5±1.4	417 (240/179)
Personal control score	4.7±2.7	4.7±2.8	4.7±2.7	416 (239/179)
Treatment control score	6.5±2.4	6.8±2.5	6.6±2.5	418 (240/178)
Identity score	6.8±1.9	6.8±2.1	6.8±2.0	420 (241/179)
Concern score	7.4±2.6	7.5±2.5	7.4±2.6	419 (240/179)
Coherence score	7.2±2.7	7.3±2.5	7.2±2.7	419 (240/179)
Emotional response score	6.4±2.7	6.5±2.8	6.4±2.7	420 (241/179)
**heiQ**				
Social engagement score	2.5±0.5	2.6±0.6	2.6±0.6	417 (237/180)
**Time Use Survey**				
Time spent doing activities over last 4 days (min)	270 (135–540)	300 (143–570)	270 (135–540)	369 (209/160)

Data are presented as median (interquartile range), n (%) or mean±sd, unless otherwise stated; percentages for categorical variables take as their denominator the number with complete data and hence sum to 100% across categories that are exclusive. mMRC: modified Medical Research Council breathlessness scale; HADS: Hospital Anxiety and Depression Scale; HADS-A: HADS anxiety subscale; HADS-D: HADS depression subscale; BDI II: Beck Depression Inventory II; BAI: Beck Anxiety Inventory; SGRQ: St George's Respiratory Questionnaire; B-IPQ: Brief Illness Perception Questionnaire; heiQ: Health Education Impact Questionnaire. ^#^: may have more than one long-term health problem; ^¶^: HADS was completed during screening prior to collection of baseline data.

### Anxiety and depression

At 6 months the mean difference between the two study arms was less than the minimal clinically important difference assumed in the sample size calculation for both co-primary outcomes (HADS-A mean difference −0.60, 95% CI −1.40–0.21; and HADS-D mean difference −0.66, 95% CI −1.39–0.07) and the limits of the 95% confidence interval effectively ruled out any clinically important effects (table 3). None of the sensitivity analyses altered the interpretation of the primary analysis, *i.e.* that the confidence interval ruled out clinically important effects. A possible exception was the analysis that excluded participants with a score <8 on HADS-A and the analysis of the intervention effect in participants attending some or all of their facilitator sessions remotely. The confidence interval in the latter case was wide (supplementary tables S3–S6). CACE analysis results were not materially different from the complete case analysis (not shown).

**TABLE 3:** Primary and secondary outcomes according to treatment arm

	**Treatment arm**		**Mean difference (95% CI)**	**p-value**	**n (data available):** **all (intervention/usual care)**
	**Intervention (n=242)**	**Usual care (n=181)**			
**HADS**					
HADS-A					
6** **months	8.09±3.85	8.94±4.19	−0.60 (−1.40–0.21)	0.145	369 (205/164)
12** **months	8.14±3.94	8.77±4.47	−0.42 (−1.25–0.40)	0.314	341 (191/150)
HADS-D					
6** **months	7.49±3.83	8.20±3.71	−0.66 (−1.39–0.07)	0.074	368 (204/164)
12** **months	8.17±4.06	8.72±4.07	−0.46 (−1.21–0.28)	0.220	342 (190/152)
**BDI II and BAI**					
BDI II					
6** **months	17.27±10.63	17.65±10.68	−0.12 (−2.16–1.91)	0.904	336 (191/145)
12** **months	16.85±10.26	17.46±10.14	−0.63 (−2.75–1.50)	0.559	288 (158/130)
BAI					
6** **months	13.96±10.32	14.55±10.76	−0.38 (−2.27–1.51)	0.692	327 (180/147)
12** **months	12.80±9.10	13.47±10.12	−0.95 (−2.92–1.01)	0.339	288 (155/133)
**SGRQ**					
Symptoms					
6** **months	58.65±22.71	58.66±24.87	−0.40 (−4.85–4.06)	0.860	352 (194/158)
12** **months	55.94±25.90	55.61±26.10	0.97 (−3.70–5.64)	0.682	210 (170/140)
Activity					
6** **months	76.00±18.40	75.37±20.00	0.02 (−3.36–3.39)	0.992	350 (193/157)
12** **months	76.18±20.62	77.70±18.14	−1.36 (−4.89–2.17)	0.446	307 (167/140)
Impact					
6** **months	42.84±19.77	44.28±19.22	−1.24 (−4.57–2.09)	0.460	347 (193/154)
12** **months	42.73±19.80	43.60±19.97	−0.15 (−3.61–3.31)	0.932	306 (165/141)
Total					
6** **months	55.50±17.23	56.04±17.34	−0.68 (−3.64–2.29)	0.652	347 (193/154)
12** **months	55.00±18.40	55.74±17.08	−0.51 (−3.61–2.59)	0.745	300 (164/136)
**B-IPQ**					
Consequences					
6** **months	6.31±2.16	6.66±2.57	−0.27 (−0.73–0.19)	0.248	349 (194/155)
12** **months	6.34±2.37	6.64±2.31	−0.29 (−0.76–0.19)	0.233	312 (172/140)
Timeline					
6** **months	9.61±1.29	9.54±1.36	0.01 (−0.28–0.30)	0.948	351 (195/156)
12** **months	9.62±1.31	9.64±1.11	−0.03 (−0.33–0.26)	0.816	312 (172/140)
Personal control					
6** **months	5.04±2.56	5.08±2.83	−0.04 (−0.65–0.57)	0.892	346 (193/153)
12** **months	5.29±2.50	5.19±2.48	0.14 (−0.49–0.77)	0.661	312 (171/141)
Treatment control					
6** **months	6.68±2.51	6.48±2.85	0.29 (−0.26–0.83)	0.299	349 (194/155)
12** **months	6.67±2.32	6.60±2.57	0.33 (−0.24–0.90)	0.258	312 (171/141)
Identity					
6** **months	6.76±2.03	6.81±2.14	−0.08 (−0.53–0.37)	0.729	348 (194/154)
12** **months	6.41±2.29	6.66±2.02	−0.23 (−0.70–0.23)	0.320	310 (170/140)
Concern					
6** **months	6.72±2.87	6.81±2.95	−0.05 (−0.56–0.46)	0.856	348 (192/156)
12** **months	6.63±2.88	7.18±2.58	−0.53 (−1.06–0.01)	0.054	312 (171/141)
Coherence					
6** **months	8.10±2.20	7.61±2.43	0.58 (0.12–1.03)	0.014	349 (194/155)
12** **months	8.20±2.09	8.18±2.20	0.12 (−0.36–0.60)	0.620	313 (172/141)
Emotional response					
6** **months	5.88±2.89	6.03±2.91	−0.22 (−0.78–0.34)	0.438	348 (194/156)
12** **months	6.12±2.76	6.13±2.92	−0.06 (−0.64–0.52)	0.833	313 (172/141)
**heiQ and Time Use Survey**					
heiQ					
6** **months	2.66±0.56	2.61±0.63	0.07 (−0.05–0.18)	0.272	352 (189/153)
12** **months	2.60±0.61	2.54±0.62	0.08 (−0.04–0.20)	0.198	301 (164/137)
Time Use Survey					
6** **months	499.9±708.2	410.8±562.2	108.5 (−52.2–269.1)	0.184	253 (149/104)
12** **months	430.2±601.4	384.7±684.3	39.2 (−149.3–227.7)	0.682	176 (97/79)

Data are presented as mean±sd, unless otherwise stated. HADS: Hospital Anxiety and Depression Scale; HADS-A: HADS anxiety subscale; HADS-D: HADS depression subscale; BDI II: Beck Depression Inventory II; BAI: Beck Anxiety Inventory; SGRQ: St George's Respiratory Questionnaire; B-IPQ: Brief Illness Perception Questionnaire; heiQ: Health Education Impact Questionnaire.

### Secondary outcomes

No statistically significant differences between the two trial arms were seen in HADS-A and HADS-D scores at 12 months nor in the other secondary outcomes, BDI II, BAI and SGRQ total and subscale scores, at either time-point, and the limits of the confidence intervals ruled out any clinically important differences (table 3). All other outcome measures, including social engagement and the Time Use Survey, were similar across participants in both arms of the study at both time-points (table 3). The prevalence of smoking fell in both arms of the study across the duration of follow-up, but there was no difference between the two arms; approximately a quarter of participants reported smoking at 12 months (table 4).

**TABLE 4:** Treatment effect on smoking at 6 and 12** **months

	**Treatment arm**		**OR (95% CI)**	**p-value**	**n (data available):** **all (intervention/usual care)**
	**Intervention (n=242)**	**Usual care (n=181)**			
**6 months follow-up**			1.11 (0.69–1.78)	0.660	360 (201/159)
Current smoker	56 (27.9)	41 (25.8)			
Nonsmoker	145 (72.1)	118 (74.2)			
**12 months follow-up**			0.90 (0.54–1.50)	0.684	321 (175/146)
Current smoker	42 (24.0)	38 (26.0)			
Nonsmoker	133 (76.0)	108 (74.0)			

Data are presented as n (%), unless otherwise stated.

In the intervention arm, 122 participants (50%) were referred to pulmonary rehabilitation by their usual healthcare providers, 112 (46%) attended at least one pulmonary rehabilitation session and 73 (30%) completed the course. In the control arm, 88 participants (49%) were referred to pulmonary rehabilitation, 77 (43%) attended at least one session and 54 (30%) completed the course. The mean±sd time to commencing pulmonary rehabilitation was 114±68.3 days in the intervention arm and 106±88.6 days in the control arm of the study.

Intervention participants received on average 4.8 intervention sessions each. Most (196 (81%)) received what we had anticipated might be the minimal clinically effective dose (two sessions) of a cognitive behavioural approach and 136 (56%) completed a course (six or more sessions) (supplementary box S5).

The results of the health economic analyses are presented in detail in the supplementary material. The mean±sd cost of delivering the TANDEM intervention was GBP 277.21±110.97 per intervention arm participant. Over 12 months the mean healthcare resource use costs were GBP 770 higher per intervention participant compared with the control arm, with a wide margin of uncertainty (95% CI GBP −27.91–1568.39).

Based on the imputed sample with adjustment for baseline covariates, TANDEM participants accumulated marginally fewer QALYs over the 12-month follow-up compared with the control participants (mean difference −0.010 QALYs, 95% CI −0.042–0.021 QALYs, equivalent to 3.7 fewer days spent in full health) (supplementary table S12).

Combining the incremental cost and incremental QALYs between the intervention and control arms, the TANDEM intervention was more expensive and less effective. The incremental net health benefit of TANDEM at the GBP 20 000 cost-effectiveness threshold was negative (−0.0489 QALYs, 95% CI −0.0512– −0.0477 QALYs) with a low probability (0.037) that TANDEM was a cost-effective alternative to usual care (supplementary table S13).

## Discussion

Following careful intervention development based on previous work [11, 15], we developed TANDEM, a novel, tailored intervention based on a cognitive behavioural approach for people with COPD with moderate to very severe airways obstruction and experiencing symptoms of mild to moderate anxiety and/or depression. We trained respiratory healthcare professionals to deliver the TANDEM intervention supervised by an experienced cognitive behavioural therapist and evaluated the intervention in a randomised controlled trial with an internal pilot. At 6 months post-randomisation the intervention had not significantly improved either of our co-primary outcomes, *i.e.* symptoms of depression or anxiety as determined by HADS-A and HADS-D. At both 6- and 12-month follow-up the intervention had not improved any of the secondary outcomes, including health status, depression and anxiety assessed using Beck instruments and social engagement, nor did it appear to influence other outcomes: uptake and completion of pulmonary rehabilitation, healthcare resource use and smoking cessation. Moreover, at both 6- and 12-month follow-up, 95% confidence intervals for estimates of effect excluded any clinically meaningful difference in our questionnaire outcome measures. The economic evaluation of the TANDEM intervention suggested that it is highly unlikely to be cost-effective. Although there were more deaths in the intervention arm than in the control arm (5% *versus* 2%), these were unrelated to the intervention and healthcare resource use was similar in both arms of the study.

Overall, our study suggests that psychological interventions with a cognitive behavioural approach do not improve symptoms of anxiety or depression in patients with advanced COPD, nor do these interventions improve disease-related quality of life or uptake or completion of pulmonary rehabilitation, although they could help with other important symptoms not assessed in this study. Despite these negative results, our study underlines the very high unmet need resulting from psychological distress in people with moderate to very severe COPD; 42% of those we screened for eligibility had symptoms of mild to moderate anxiety or depression.

This is by far the largest randomised controlled trial of any psychological intervention for people with COPD, and one of the few to include only those with symptoms of anxiety and/or depression at baseline [5]. We are aware of seven previously published studies which evaluated cognitive behavioural therapy/approaches in participants with COPD and anxiety or depressive symptoms at baseline in randomised controlled trials [29–35]: four were very small and underpowered and/or at very high risk of bias [27, 29, 30, 32], and two had attrition >40% in one or both study arms [29, 31]. The largest study (n=279) looked at participants with COPD and symptoms of anxiety, and reported lower HADS-A scores at 3 months post-intervention in those receiving brief cognitive behavioural therapy compared with the control arm. This difference diminished and was no longer clinically significant at 6- or 12-month follow-up, but overall the intervention was cost-effective [34].

A Cochrane review of psychological therapies for the treatment of anxiety in COPD identified only three studies, all of which included patients with anxiety and depression [36]. These three studies were also included in the Cochrane review of psychological interventions for people with COPD and depression [5]. This review concluded: “… psychological therapies (using a [cognitive behavioural therapy]-based approach) may be effective for treating COPD-related depression, but the evidence is limited. … the effect sizes were small and quality of the evidence very low due to clinical heterogeneity and risk of bias” [5]. Several of the included studies had high levels of attrition. The review called for larger, more robust studies that consider adverse events, health service use and cost-effectiveness outcomes, all of which we addressed in our trial.

Our study was adequately powered with very good retention at 12-month follow-up. Allocation was fully concealed and participants were randomised after collection of baseline data. Although we could not mask the participants or facilitators to allocation, all healthcare professionals involved in their care were unaware of participants’ allocation. Outcome assessors were masked and accidental unmasking was rare. Trial statisticians were masked prior to the statistical analysis plan being signed off and the database being locked. Patient and Public Involvement colleagues were involved throughout the trial [37]. The intervention appeared acceptable as 80% of participants received our predetermined minimal clinically effective dose and 56% (136) received a complete course. These figures compare favourably to the overall uptake and completion rates for the Increasing Access to Psychological Therapies service in England [38]. We assessed fidelity of intervention delivery and the acceptability of the intervention to stakeholders and conducted a detailed process evaluation [17, 39, 40]. In summary: fidelity of intervention delivery and intervention acceptability were generally high.

The trial had some limitations. Only half of the nearly 4500 people contacted by their clinical team as potentially eligible responded. Although written English was not a requirement, we required sufficient fluency in English to consent to the study and to participate in the intervention. Our recruitment sites covered areas with geographically and ethnically diverse populations, but we did not formally collect participants’ ethnicity so are unable to comment on the mix within our study.

The question arises why the TANDEM intervention was not effective. It may be difficult for any relatively brief cognitive behavioural intervention to influence psychological comorbidities that have developed alongside a multicomponent condition which has developed across the life course [1], especially coming late in the trajectory of many participants’ COPD. Overall, our trial participants were disabled by their COPD and had low baseline health-related quality of life. Most suffered from other comorbid health conditions and, anecdotally, several had difficult, complex life situations or were also themselves carers. Ultimately our carefully designed intervention may have been “too little, too late” for TANDEM participants experiencing all the attendant difficulties of living with a disabling long-term condition and comorbidity. It is also possible that it was challenging for our healthcare professional facilitators to deliver the cognitive behavioural approach effectively, although they received structured, expert supervision throughout and formal assessment of fidelity suggested that overall, they delivered the intervention well [17]. There is evidence from other literature that trained healthcare professionals can effectively deliver psychological interventions to people with long-term conditions [41], including COPD [5, 34]. We set our primary outcome at 6 months because we hoped that our intervention would make a sustained change to participants’ lives. It is possible that had we measured outcomes at the end of intervention delivery, or at 3 months, we might have seen transient evidence of benefit of the intervention.

This trial indicates that cognitive behavioural interventions may not improve symptoms of anxiety or depression in patients with advanced COPD. Further research is needed to develop and evaluate alternative approaches to relieve the disease burden for this patient group. We suggest that three different approaches should be investigated. First, respiratory healthcare professional-delivered psychological approaches might be effective if delivered much earlier in the trajectory of this condition and could then make a lasting change to patients. Second, although pulmonary rehabilitation is known to improve mood and confers many other benefits for people with COPD, we found only 50% of those eligible for referral to pulmonary rehabilitation were actually referred and only 30% of those who attended completed a course. There is an urgent need to identify why patients are not being referred to pulmonary rehabilitation. Alongside this we need more research around supporting individuals to take up and complete a course of pulmonary rehabilitation, and we need to identify effective, alternative interventions for those individuals who will never be able to attend conventional pulmonary rehabilitation. Finally, we have identified considerable unmet need in a group of people with advanced COPD. There should be research in how to support this group more effectively, *e.g.* looking at the role of assistive technology.

## Supplementary material

10.1183/13993003.00432-2023.Supp1 **Please note:** supplementary material is not edited by the Editorial Office, and is uploaded as it has been supplied by the author.
Supplementary material 1: supplementary tables and boxes ERJ-00432-2023.Supplement
Supplementary material 2: TANDEM Study statistical analysis plan ERJ-00432-2023.Supplement_2

## Shareable PDF

10.1183/13993003.00432-2023.Shareable1 This one-page PDF can be shared freely online.
Shareable PDF ERJ-00432-2023.Shareable
