# Supplementary material for: Tailored psychological intervention for anxiety or depression in COPD (TANDEM): a randomised controlled trial
Source: Eur Respir J. 2023 Nov 2;62(5):2300432. doi: 10.1183/13993003.00432-2023 (PMC10620475; doi:10.1183/13993003.00432-2023)
Supplement: Supplementary file 1 [file ERJ-00432-2023.Supplement.pdf]

**Tailored psychological intervention for anxiety or depression in COPD (TANDEM): a randomised controlled trial**

**Supplementary Materials 1**

**Table of contents**


| **Supplementary component** | **Page no.** |
|---|---|
| Table 1. Changes made to the protocol over the course of the trial | 2 |
| Box1. Description of TANDEM Facilitators. | 3 |
| Table 2. Further information on study outcomes | 3-4 |
| Box 2. Details of anomaly in randomisation ratio and additional sensitivity analyses conducted | 4 |
| Box 3. Health economic analysis methods | 5 |
| Box 4. Impact of the COVID-19 pandemic on the Trial | 5 |
| | |
| ***Results of sensitivity analyses*** | 6-9 |
| Table 3. Results from sensitivity analyses for HADS-A at 6 months | 6 |
| Table 4. Results from sensitivity analyses for HADS-D at 6 months | 6-7 |
| Table 5. Results of sensitivity analyses for data being missing not at random (MNAR): HADS-A at 6 months | 7-8 |
| Table 6. Results of sensitivity analyses for data being missing not at random (MNAR): HADS-D at 6 months | 8 |
| Table 7. Baseline patient characteristics according to whether 6-month data collection was pre-pandemic or during the pandemic and according to the mode of intervention delivery. | 9-10 |
| | |
| Box 5. Details of the number of facilitator sessions delivered to participants and rationale for minimum clinically effective dose | 11 |
| Figure 1. Number of TANDEM Facilitator sessions delivered to participants. | 11 |
| | |
| ***Health economics analysis*** | 12- |
| Box 6. Cost of implementing the TANDEM programme | 12 |
| Table 8. Mean cost of delivering the TANDEM intervention | 12 |
| Table 9. TANDEM training costs | 12-13 |
| Table 10. Service use for observed data for participants in both study arms at 12 months | 13 |
| Table 11. Mean cost for observed data (£) by resource category by randomised group over the 12-months follow-up. | 13 |
| Table 12. Mean participant total healthcare resource use cost and quality adjusted life years (QALYs) | 14 |
| Figure 2. Cost-effectiveness plane (base case results) | 14 |

| | |
|---|---|
| Figure 3. Cost-effectiveness acceptability curve (base case results) | 15 |
| Table 13. Estimate of incremental net health benefit (INHB) for different cost-effectiveness thresholds (base case results) | 15 |
| Box 7: Health economics sensitivity analyses | 15 |
| Supplementary Table 14. Sensitivity analysis: results after trimming outliers | 16 |
| Supplementary Table 15. Sensitivity analysis: results after using alternative estimation of training | 16 |
| References | 16-17 |


**Supplementary Table 1. Changes made to the protocol over the course of the trial**

| Document date and protocol version number[1] | Details of amendment[1] |
|---|---|
| 04/Apr/2017<br>Version 2·0 | The change proposed was only at the randomisation level. The decision was made that the randomisation would be performed by stratification by NHS Trust, and using minimisation within each stratum with a random element, balanced for important patient characteristics to ensure treatment groups are well matched at baseline |
| 13/Jun/2017<br>Version 3·0 | Update of recruitment strategy i.e. producing a new letter for approved study leaflet and clinical teams being able to approach patients who have previously declined pulmonary rehabilitation |
| 17/Aug/2017<br>Version 4·0 | Addition of 'very severe COPD' and 'FEV1 predicted <80%', FEV1 predicted <30-80%' phrase where appropriate. |
| 22/Apr/2018<br>Version 6·0 (approved in sub-committee meeting) following submission of 5·0 for approval | Refinements and revisions to the study protocol and study documentation to be used in the main trial based on learning from the pilot and the addition of new study sites. The revisions mostly include changes in the form of clarifications that would be used by the research team to operationalise the protocol. One change in the study documentation related to informing and requesting consent from participants about contacting them in five years following completion of the study to assess their long-term quality of life/survival. |
| 29/May/2019<br>Version 8·0 (approved in sub-committee meeting) following submission of 7·0 for approval | Collection of health care use data from NHS Digital is part of the approved study protocol (v8.0 29 May 2019). To operationalise this, we sought written permission from study participants. In addition, a brief statement has been added in the Screening results letter to ensure safety of study team members doing fieldwork (study visits conducted in patient homes). The application to use NHS Digital was eventually withdrawn because of delays by NHS Digital in reviewing the application. |
| 06/Mar/2020<br>Version 9·0 | Extending the study timeline, following approval from funder (current study timeline is 30 June 2020) by 12 months with study due for completion by 30 June 2021. Reason for the extension is to complete patient recruitment by March-end 2020) and then completion of 6 month and 12 month follow up assessments and analysis. |

[1]. All protocol changes underwent ethics and governance approvals.

**Box 1. Description of TANDEM Facilitators**

- The 31 trained Facilitators delivering the intervention included: nurse specialists (n=7); physiotherapists (n=16); occupational therapists (n=6), one health psychologist and one exercise physiologist.
- To avoid contamination and preserve masking of healthcare providers, TANDEM Facilitators were not involved in the provision of routine COPD care, or the delivery of PR, at participating sites.
- Typically, Facilitators were working part time in neighbouring NHS organisations not involved in the study.
- Facilitators were trained over three days across six weeks and assessed using a simulated patient (an actor) at the end of training.

**Table 2. Further information on study questionnaire outcomes**

| Outcome measure | Description, rationale | MCID[1], if known |
|---|---|---|
| Hospital Anxiety and Depression Scale (HADS) | A 14 item questionnaire with two subscales: seven questions relate to symptoms of anxiety (HADS-A subscale), and seven questions relate to symptoms of depression (HADS-D subscale). Participants score between 0-21 on each subscale, higher scores indicate worse symptoms. Validated cut off points for probable mild anxiety or depression ≥ 8 or more on each subscale), for severe anxiety or depression >15.[2] Using ≥8 as a cut off for anxiety/depression, the HADS has high sensitivity (80%) and specificity (90%),[3] although, probably in common with most single instruments, it does not provide good separation between symptoms of anxiety and depression.[4]<br><br>HADS is commonly used in studies of COPD, specifically in the context of CBT interventions for breathlessness in COPD,[5,6] and pulmonary rehabilitation for anxiety and depression.[7,8] | 1.5[1] |
| Beck Depression Inventory II (BDI II)[9] | The Beck Depression Inventory II (BDI II)[9]is widely used in depression trials.[10] Participants score from 0 to 63 with higher scores being more depressed. BDI II scores in the range 14-19 indicate mild depression and 20-28 suggests moderate depression.[11] | 5 [12] or 3[10] although the latter has been challenged (may be reasonable in this trial as baseline BDI 20.4).[10]<br><br>Not established for a population of people with COPD. |
| Beck Anxiety Inventory (BAI)[13] | The Beck Anxiety Inventory (BAI) is widely used in psychiatric research.[13,14] Participants score from 0 to 63 with higher scores indicating worse symptoms. BAI scores of 21 or lower suggest low anxiety and scores from 21 to 35 suggest moderate anxiety.[13] Measures the severity of anxiety in primary care studies but may not discriminate well between symptoms of anxiety and depression in primary care studies.[15] | Unclear |
| St George's | The SGRQ measures Health-related quality-of-life in people with | 4 for the |

| | | |
|---|---|---|
| Respiratory Questionnaire (SGRQ)[16] | chronic respiratory disease, particularly COPD. It has three subscales (Symptoms, Activity and Impacts) and a Total score.[17] The Total score is computed out of 100 which represents the percentage of overall impairment where 100 represents the worst overall possible health status. | Total score[18] |
| Social Integration and Support subscale of the Health Education Impact Questionnaire (heiQ)[19] | This subscale has 5 items, participants score responses to each item on a 4-point Likert scale, from "strongly disagree/strongly agree". The subscale score is the mean of the responses higher scores indicate stronger skills in the management of the chronic condition. | N/a |
| An adapted version of the UK Time Use Survey[20] | Social activity was measured using an adapted version of the UK Time Use Survey,[20] which asks how many times, and for how long, the interviewee had engaged in various social activities activity in the previous week and whether this was alone or with another person.[21] | N/a |
| Brief Illness Perception Questionnaire (B-IPQ)[22] | Brief Illness Perception Questionnaire (B-IPQ), a nine-item questionnaire which examines the cognitive and emotional representations an individual holds of their illness,[22] included to understanding how the intervention might be working. | N/a |

[1] Minimal clinically important difference, N/a = not available for populations with COPD

**Box 2. Details of Anomaly in allocation ratio and additional sensitivity analyses conducted**

During the trial, an anomaly in the allocation ratio produced by the online randomisation system became apparent. Specifically, over the first 69 randomisations the observed allocation ratio was around 1.25:1 as expected, but over the next 70 randomisations the observed allocation ratio was around 5:1. The deviation from the expected allocation ratio during this second period of randomisations triggered a Corrective Action and Prevention plan overseen by the sponsor and with the approval of the Data Monitoring and Ethics Committee, that included migrating the randomisation system to a new platform. For the remainder of randomisations an allocation ratio of 1:1 was specified in order to return the overall allocation ratio to a figure closer to 1.25:1, and the observed allocation ratio over this third randomisation period was close to 1:1.

- As an additional sensitivity analysis for the two primary outcomes we included randomisation period (period 1, period 2, or period 3) as an additional covariate (see Supplementary materials Tables 3 & 4, below).
- We also present a table of baseline characteristics of participants in different randomisation periods (and in different trial arms) to confirm the balance of characteristics and to allow an exploration of whether these characteristics might have differed in different periods (though since the trial recruited fairly rapidly we did not expect this to be the case) (see Supplementary materials Table 7, below).

**Box 3. Health economic analysis methods**

Intervention costs were calculated using a combination of data from patients' general practice records and a modified version of the Client Service Receipt Inventory.[23] The general practice records data acted as the primary source of information on health service contacts with the self-reported data used to supplement the service use data with details of service utilisation not covered by the GP records. Health and social care utilisation were costed using applicable unit costs derived from national NHS Reference Costs and the Unit Costs of Health and Social Care.[24] The evaluation adopted a "cost-utility" framework, with the incremental resource impact of TANDEM over usual care quantified from an NHS/Personal Social Services perspective and patient outcomes quantified as incremental quality-adjusted life years (QALYs) gained. QALYs over 12-month follow-up were estimated based on the EQ-5D-5L instrument. Scores applicable to the EQ-5D-5L were "cross walked" to their equivalent 3-level version values as recommended.[25,26]

QALY differences were estimated using a mixed effects random intercepts model; total cost difference was estimated using a mixed-effects generalised linear modelling specification (Gamma family, log link function). Intervention cost-effectiveness was evaluated with reference to the incremental net health benefit (INHB) of TANDEM combined with usual care compared to usual care alone (INHB expressed in QALY units) and estimated assuming a cost-effectiveness threshold of £20,000 per QALY gained (this threshold was varied in sensitivity analyses). Uncertainty due to sampling error was quantified through non-parametric bootstrap sampling from the trial data. Multiple imputation was used to replace missing data assuming data were missing at random (MAR).

**Box 4. Impact of the COVID-19 pandemic on the Trial**

- We were instructed by our Research Management Office to cease recruitment 0n 19th March 2020 due to the COVID pandemic. At this point we had recruited and randomised 425 participants and we had also identified several other potentially eligible patients who were willing to be screened for eligibility. Without the pandemic we would have achieved our planned sample size, 430 participants.
- The pandemic disrupted face-to-face delivery of the intervention for the final 25 (10%) intervention participants, including six who had commenced the intervention face-to-face. We moved to remote delivery of TANDEM sessions; 18/25 participants (72%) completed intervention delivery.
- After mid-March 2020 all participant questionnaire follow-up data were collected by phone or by post. During the pandemic researchers were unable to visit general practices to collect data from primary care records greatly reducing the health service usage data we were able to obtain after March 2020.

## Results of sensitivity analyses

**Table 3. Results from sensitivity analyses for HADS-A at 6 months**

| | Treatment effect | 95% CI | p-value |
|---|---|---|---|
| Main analysis | -0·60 | (-1·40; 0·21) | 0·145 |
| Complete case analysis | -0·69 | (-1·48; 0·11) | 0·090 |
| Missing items imputed for participants with partially incomplete HADS-A | -0·61 | (-1·41; 0·19) | 0·137 |
| Excluding participants with a score <8 on HADS-A subscale | -0·95 | (-1·88; -0·01) | 0·048 |
| Time to PR as an additional covariate | 0·60 | (-1·40; 0·20) | 0·143 |
| Excluding internal pilot participants | -0·50 | (-1·35; 0·35) | 0·246 |
| Randomisation period as an additional covariate | -0·60 | (-1·41; 0·21) | 0·144 |
| Pre- *vs* during pandemic as an additional covariate | -0·63 | (-1·45; 0·19) | 0·130 |
| Pre- *vs* during pandemic as a treatment effect modifier | | | 0·565 [a] |
| Pre-pandemic | -0·72 | (-1·59; 0·16) | |
| During pandemic | -0·33 | (-1·62; 0·96) | |
| Fully face-to-face *vs* remote delivery *vs* no CBA as additional covariate | 0·52 | (-1·07; 2·12) | 0·519 |
| Fully face-to-face *vs* remote delivery *vs* no CBA as a treatment effect modifier | | | 0·606 [a] |
| Fully face-to-face | -0·11 | (-0·91; 0·70) | |
| Partially or fully remote | -0·85 | (-2·40; 0·70) | |
| No CBA | 0·61 | (-1·14; 2·36) | |

[a] p-value for interaction

**Table 4. Results from sensitivity analyses for HADS-D at 6 months**

| | Treatment effect | 95% CI | p-value |
|---|---|---|---|
| Main analysis | -0·66 | (-1·39; 0·07) | 0·074 |
| Complete case analysis | -0·77 | (-1·51; -0·03) | 0·041 |
| Missing items imputed for participants with partially incomplete HADS-D | -0·70 | (-1·42; 0·02) | 0·058 |
| Excluding participants with a score <8 on HADS-D subscale | -0·66 | (-1·52; 0·20) | 0·129 |
| Time to PR as an additional covariate | -0·64 | (-1·35; 0·08) | 0·079 |
| Excluding internal pilot participants | -0·63 | (-1·44; 0·17) | 0·120 |
| Randomisation period as an additional covariate | -0·65 | (-1·37; 0·07) | 0·078 |
| Pre- *vs* during pandemic as an additional covariate | -0·64 | (-1·35; 0·08) | 0·082 |

| | | | |
|---|---|---|---|
| Pre- *vs* during pandemic as a treatment effect modifier | | | 0·867 |
| Pre-pandemic | -0·66 | (-1·43; 0·11) | |
| During pandemic | -0·56 | (-1·71; 0·58) | |
| Fully face-to-face *vs* remote delivery *vs* no CBA as additional covariate | 0·37 | (-1·15; 1·88) | 0·634 |
| Fully face-to-face *vs* remote delivery *vs* no CBA as a treatment effect modifie | | | 0·634 |
| Fully face-to-face | -0·29 | (-0·96; 0·39) | |
| Partially or fully remote | -0·05 | (-1·47; 1·37) | |
| No CBA | 0·68 | (-0·98; 2·33) | |

[a] p-value for interaction

**Table 5. Results of sensitivity analyses for data being missing not at random (MNAR): HADS-A at 6 months**

| Assumed difference in mean responses for participants with missing data compared with participants with complete data | | | |
|---|---|---|---|
| **Usual care** | **Intervention** | **Treatment effect** | **95% CI** |
| -10 | -15 | -2·04 | (-2·83, -1·25) |
| | -10 | -1·28 | (-2·06, -0·49) |
| | -5 | -0·51 | (-1·30, 0·28) |
| -5 | -10 | -1·75 | (-2·53, -0·96) |
| | -5 | -0·98 | (-1·77, -0·19) |
| | 0 | -0·22 | (-1·00, 0·57) |
| -1·5 | -6·5 | -1·54 | (-2·33, -0·75) |
| | -1·5 | -0·78 | (-1·56, 0·01) |
| | 3·5 | -0·01 | (-0·80, 0·78) |
| 0 | -5 | -1·45 | (-2·24, -0·66) |
| | 0 | -0·69 | (-1·47, 0·10) |
| | 5 | 0·08 | (-0·71, 0·86) |
| 1·5 | -3·5 | -1·36 | (-2·15, -0·58) |
| | 1·5 | -0·60 | (-1·39, 0·19) |
| | 6·5 | 0·17 | (-0·62, 0·95) |
| 5 | 0 | -1·16 | (-1·94, -0·37) |
| | 5 | -0·39 | (-1·18, 0·40) |

| | | | |
|---|---|---|---|
| | 10 | 0·37 | (-0·41, 1·16) |
| 10 | 5 | -0·86 | (-1·65, -0·07) |
| | 10 | -0·10 | (-0·88, 0·69) |
| | 15 | 0·67 | (-0·12, 1·45) |

**Table 6. Results of sensitivity analyses for data being missing not at random (MNAR): HADS-D at 6 months**

| Assumed difference in mean responses for participants with missing data compared with participants with complete data | | | |
|---|---|---|---|
| **Usual care** | **Intervention** | **Treatment effect** | **95% CI** |
| -10 | -15 | -2·19 | (-2·92, -1·46) |
| | -10 | -1·40 | (-2·13, -0·67) |
| | -5 | -0·62 | (-1·35, 0·11) |
| -5 | -10 | -1·87 | (-2·60, -1·14) |
| | -5 | -1·09 | (-1·82, -0·36) |
| | 0 | -0·30 | (-1·03, 0·43) |
| -1·5 | -6·5 | -1·65 | (-2·38, -0·92) |
| | -1·5 | -0·87 | (-1·60, -0·14) |
| | 3·5 | -0·08 | (-0·81, 0·65) |
| 0 | -5 | -1·56 | (-2·29, -0·83) |
| | 0 | -0·77 | (-1·50, -0·04) |
| | 5 | 0·01 | (-0·72, 0·74) |
| 1·5 | -3·5 | -1·46 | (-2·19, -0·73) |
| | 1·5 | -0·68 | (-1·41, 0·05) |
| | 6·5 | 0·11 | (-0·62, 0·84) |
| 5 | 0 | -1·16 | (-1·94, -0·37) |
| | 5 | -0·39 | (-1·18, 0·40) |
| | 10 | 0·37 | (-0·41, 1·16) |
| 10 | 5 | -0·86 | (-1·65, -0·07) |
| | 10 | -0·10 | (-0·88, 0·69) |
| | 15 | 0·67 | (-0·12, 1·45) |

Table 7. Baseline patient characteristics according to whether 6-month data collection was pre-pandemic or during the pandemic and according to the mode of intervention delivery[a]

| | Six month data collected before 19 March 2020 | | | | Six month data collected after 19 March 2020 | | | | Fully face-to-face delivery | | Partly remote delivery | |
|---|---|---|---|---|---|---|---|---|---|---|---|---|
| | Intervention | | Usual care | | Intervention | | Usual care | | | | | |
| **Participant demographics** | (n=162) | | (n=119) | | (n=44) | | (n=44) | | (n=195) | | (n=22) | |
| Age (years) - Median (IQR) | 68 | (61,76) | 70 | (63, 74) | 69 | (62, 76) | 65 | (59, 74) | 69 | (62, 77) | 68 | (62.5, 74) |
| Gender - No. (%) | | | | | | | | | | | | |
| Male | 78 | *48.1%* | 55 | *46.6%* | 30 | 68.2% | 17 | *38.6%* | 104 | 53.3% | 15 | 68.2% |
| Female | 84 | *51.9%* | 63 | *53.4%* | 14 | 31.8% | 27 | *61.4%* | 91 | 46.7% | 7 | 31.8% |
| Smoking status - No. (%) | | | | | | | | | | | | |
| Current smoker | 46 | *28.4%* | 38 | *31.9%* | 13 | *29.5%* | 9 | *20.5%* | 53 | 27.2% | 7 | 31.8% |
| Ex-smoker | 111 | *68.5%* | 80 | *67.2%* | 30 | *68.2%* | 35 | *79.5%* | 137 | 70.3% | 14 | 63.6% |
| Never smoked | 5 | *3.1%* | 1 | *0.8%* | 1 | *2.3%* | 0 | *0.0%* | 5 | 2.6% | 1 | 4.5% |
| **Degree of breathlessness (mMRC breathlessness scale) - No. (%)** | | | | | | | | | | | | |
| • Not troubled by breathlessness except on strenuous exercise | 2 | *1.2%* | 0 | *0.0%* | 0 | *0.0%* | 0 | *0.0%* | 2 | *1.0%* | 1 | *4.5%* |
| • Short of breath when hurrying on the level or walking up a slight hill | 31 | *19.1%* | 24 | *20.2%* | 6 | *13.6%* | 6 | *13.6%* | 37 | *19.0%* | 3 | *13.6%* |
| • Walks slower than other people of the same age on the level | 54 | *33.3%* | 34 | *28.6%* | 15 | *34.1%* | 19 | *43.2%* | 64 | *32.8%* | 6 | *27.3%* |
| • Stops for breath after walking about 100 yards or after a few minutes on the level | 54 | *33.3%* | 38 | *31.9%* | 14 | *31.8%* | 11 | *25.0%* | 61 | *31.3%* | 6 | *27.3%* |
| • Too breathless to leave the house or breathless when dressing or undressing | 21 | *13.0%* | 23 | *19.3%* | 9 | *20.5%* | 8 | *18.2%* | 31 | *15.9%* | 6 | *27.3%* |
| • **HADS – Mean (SD)** | | | | | | | | | | | | |
| HADS-A total score | 9.5 | (3.2) | 9.8 | (3.1) | 10.3 | (2.6) | 10.5 | (3.5) | 9.6 | (3.2) | 9.6 | (2.3) |

| | | | | | | | | | | | | |
|---|---|---|---|---|---|---|---|---|---|---|---|---|
| HADS-D total score | 9.1 | (3.1) | 9.0 | (3.0) | 9.3 | (3.0) | 9.1 | (3.3) | 9.1 | (3.0) | 8.7 | (3.9) |
| **BDI-II – Mean (SD)** | | | | | | | | | | | | |
| BDI total score | 19.9 | (8.8) | 21.0 | (10.4) | 20.3 | (8.2) | 19.6 | (10.1) | 19.6 | (8.4) | 20.6 | (10.7) |
| BAI total score | 16.5 | (10.7) | 16.1 | (10.0) | 16.9 | (9.7) | 19.2 | (9.9) | 16.5 | (10.2) | 16.1 | (11.2) |
| **SGRQ – Mean (SD)** | | | | | | | | | | | | |
| Total score | 58.2 | (15.0) | 58.1 | (15.4) | 63.5 | (15.1) | 60.8 | (15.3) | 59.4 | (14.9) | 56.8 | (19.3) |
| Symptoms | 61.3 | (20.8) | 61.5 | (24.4) | 71.0 | (19.7) | 65.5 | (22.0) | 63.8 | (21.0) | 63.8 | (19.9) |
| Activity | 77.4 | (18.1) | 76.8 | (15.6) | 83.5 | (16.3) | 81.1 | (13.4) | 78.1 | (18.3) | 76.9 | (23.3) |
| Impact | 46.3 | (16.8) | 46.4 | (17.9) | 49.7 | (16.7) | 47.7 | (19.2) | 47.4 | (16.5) | 43.2 | (21.4) |
| **B-IPQ – Mean (SD)** | | | | | | | | | | | | |
| Consequences | 6.3 | (2.2) | 6.6 | (2.3) | 6.4 | (2.0) | 6.6 | (2.3) | 6.4 | (2.1) | 6.3 | (2.6) |
| Timeline | 9.5 | (1.4) | 9.4 | (1.5) | 9.7 | (0.8) | 9.3 | (1.7) | 9.5 | (1.4) | 9.9 | (0.5) |
| Personal control | 4.8 | (2.7) | 4.7 | (2.8) | 3.9 | (2.7) | 4.7 | (2.6) | 4.7 | (2.7) | 5.0 | (2.5) |
| Treatment control | 6.5 | (2.5) | 6.9 | (2.2) | 6.3 | (2.2) | 6.7 | (2.8) | 6.4 | (2.4) | 6.8 | (2.6) |
| Identity | 6.7 | (2.0) | 6.8 | (2.2) | 7.1 | (1.7) | 6.6 | (1.8) | 6.8 | (1.9) | 6.6 | (1.9) |
| Concern | 7.2 | (2.7) | 7.4 | (2.6) | 7.8 | (2.2) | 7.7 | (2.3) | 7.4 | (2.5) | 7.4 | (2.9) |
| Coherence | 7.3 | (2.6) | 7.3 | (2.6) | 6.6 | (3.2) | 7.0 | (3.0) | 7.3 | (2.7) | 7.5 | (2.7) |
| Emotional response | 6.3 | (2.9) | 6.3 | (2.9) | 6.9 | (1.7) | 6.8 | (2.7) | 6.3 | (2.7) | 6.1 | (2.1) |
| **heiQ – Mean (SD)** | | | | | | | | | | | | |
| heiQ social engagement | 2.6 | (0.5) | 2.6 | (0.6) | 2.6 | (0.6) | 2.6 | (0.6) | 2.6 | (0.5) | 2.5 | (0.7) |
| **Time use survey – Median (IQR)** | | | | | | | | | | | | |
| Time (mins) spent doing activities over last 4 days | 335 | (160, 570) | 330 | (150, 630) | 240 | (120, 420) | 300 | (180, 480) | 270 | (135, 540) | 190 | (120, 420) |

[a] Either number (percentage), mean (standard deviation (SD)) or median (interquartile range (IQR)), as indicated in the first column. Percentages for categorical variables take as their denominator the number with complete data, and hence sum to 100% across categories that are exclusive.

**Box 5. Details of the number of facilitator sessions delivered to participants and rationale for minimum clinically effective dose**

**Figure 1. Number of TANDEM Facilitator sessions delivered to participants**

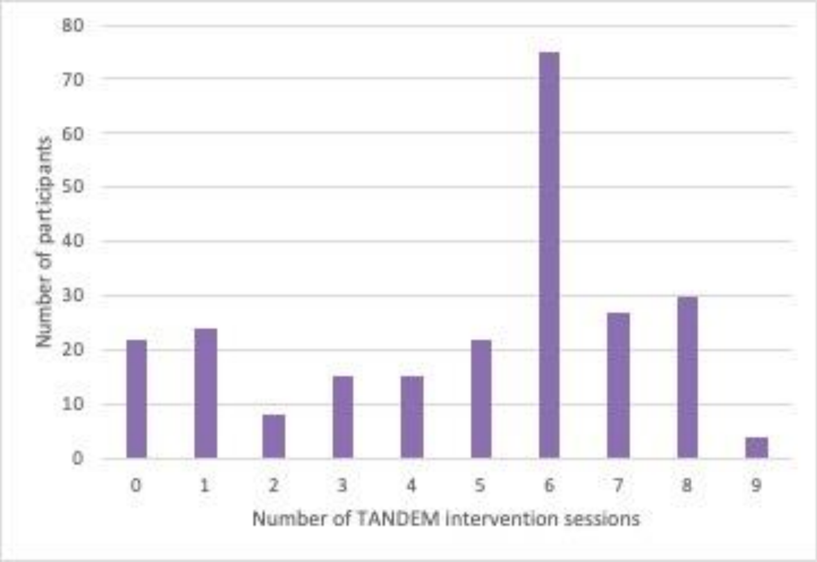


The intervention was designed to be 6 to 8 sessions tailored to the participant's need, for example if the participant had significant anxiety *and* depression they might require 8 sessions. In addition, Facilitators could repeat sessions if they were interrupted by illness or hospitalisation. We chose two sessions as our anticipated minimally clinically effective dose of intervention whilst developing the intervention because we were aware that in some cases improvement has been reported following two sessions of a psychological treatment.[27]

**Health economics analysis**

**Box 6. Cost of implementing the TANDEM programme**

For participants in the intervention arm of the trial the number and length of therapeutic contacts with TANDEM Facilitators were obtained from clinical report forms completed by the Facilitators for each individual participant, see Supplementary materials Table 8, below. Data on the frequency of participant attendance at pulmonary rehabilitation programmes were extracted at 12 months post-randomisation from pulmonary rehabilitation service case records. Implementation of the TANDEM programme also requires investment in the training of Facilitators and time allocated to the clinical psychologists' supervisory activity. To cost training activities the TANDEM project team provided the following data: the number and length of TANDEM training sessions held; the number of TANDEM Facilitators attending each training session and the clinical grades of those attending and running the training sessions; and costs of accommodation and travel. Similarly, to cost supervision, information was provided on the time used to train the supervisors and the clinical time allocated by supervisors and Facilitators to supervisory activity linked to TANDEM.

To calculate the training cost per participant we estimated the potential annual treatment caseload for the trained TANDEM Facilitators (42 in total) based on extrapolation of the number of

participants in the intervention arm of the trial, assuming a completed episode of the intervention would take 8 weeks (the maximum anticipated length of a completed course) and 42.6 working weeks over a year. See Supplementary materials Table 9, below for details of the assumptions used to estimate training and supervision costs.

**Table 8. Mean cost of delivering the TANDEM intervention**

| Variable | Mean | SD | Median | IQR | N (data available) |
|---|---|---|---|---|---|
| Number of TANDEM sessions (session) | 4·77 | 2·59 | 6 | 3 to 6 | 242* |
| Cost of sessions (£) | 205·8 | 110·97 | 256·55 | 128·48 to 266·14 | 242 |
| Training time (hour) | 29·53 | 35 | 21·75 | 21·5 to 24·5 | 42** |
| Training cost without supervision (£) | 1580·95 | 2351·04 | 1008·63 | 859·39 to 1137·18 | 42 |
| Training cost with cost of supervision (£) | 1878·06 | 2982·73 | 1017·1 | 859·39 to 1171·86 | 42 |
| Training cost per case (£) | 71·41 | - | - | - | - |
| Total cost of delivering TANDEM per programme participant, £ sterling | 277·21 | 110·97 | 327·96 | 199·89 to 337·55 | 242 |

*Number of participants in the intervention arm; **Number of healthcare professionals involved in the training·

**Table 9. TANDEM training costs**

| Costs and unit estimation | 2020/2021 value | Notes |
|---|---|---|
| Hourly rate | £23·44 | Based on the median salaries for Agenda for Change (AfC) bands· |
| Salary oncosts | £8·21 | Based on the NHS contribution rate for the period 1 April 2019 to 31 March 2023 of 20·6 per cent of pensionable pay for both the 1995-2008 Scheme and the 2015 Scheme, and 13·8 per cent employer national insurance rates for the 2021 to 2022 tax year |
| Overheads | £14·77 | Management and other non-care staff are 24·5 per cent of direct care salary costs and include administration and estates staff, section 9 PSSRU 2020· Non-staff costs are 38·2 per cent of direct care salary costs· They include costs to the provider for office, travel/transport, publishing, training courses and conferences, supplies and services (clinical and general), and utilities such as water, gas and electricity |
| Training hours | 21·75 | Based on the median hours of receiving and delivering the training |
| Total training cost | £92,025·18 | This is the cost of training 42 facilitators· It includes the opportunity cost of attending a training session, the hourly cost of delivering a training session and the cost of clinical supervision (£14,558·61) |

| | | |
|---|---|---|
| Caseload | 1288·65 | This is the expected number of cases per working year· The facilitators in the study dealt with 242 cases during 8 weeks of the intervention period· The working weeks per year are 42·6· That is 37·5 hours per week minus annual, 8 statutory leave days, days sickness leave and study/training days· |
| Cost per case | £71·41 | |

**Table 10. Service use for observed data (unit) at 12 months (for previous twelve months)**

| | | Trial arm | | | | *n* (data available) |
|---|---|---|---|---|---|---|
| **Resource** | **Unit** | **Intervention** ***n*= 242** | | **Usual care** ***n*=181** | | **All** (Intervention; usual care) |
| **Hospital-based health and social care – Mean (SD)** | | **Mean (SD)** | | | | |
| Inpatient stay | Bed days | 2·20 | 6·20 | 2·71 | 10·81 | 338 (192,146) |
| Accident and emergency not admitted | Attendances | 0·32 | 0·62 | 0·33 | 0·69 | 338 (192,146) |
| Accident and emergency admitted | Attendances | 0·31 | 0·73 | 0·39 | 1·07 | 338 (192,146) |
| Hospital outpatient clinic | Appointments | 2·53 | 2·14 | 2·14 | 2·04 | 338 (192,146) |
| **Community-based health and social care – Mean (SD)** | | **Mean (SD)** | | | | |
| GP (surgery) | Attendance | 5·08 | 5·24 | 5·12 | 5·98 | 338 (192,146) |
| Nurse (surgery) | Attendance | 4·24 | 7·94 | 3·26 | 3·97 | 338 (192,146) |
| Counselling/Therapy | Session | 0·12 | 0·60 | 0·38 | 1·37 | 338 (192,146) |
| Stop smoking service | Session | 0·29 | 1·40 | 0·11 | 0·53 | 338 (192,146) |
| Other community health-care services | n/a | 2·12 | 6·62 | 1·28 | 2·99 | 338 (192,146) |

**Table 11. Mean cost for observed data (£) by resource category by randomised group over the 12-months follow-up·**

| | **Intervention** ***n*= 242** | | **Usual care** ***n*=181** | | *n* (data available) |
|---|---|---|---|---|---|
| **Resource category** | **Mean** | **SD** | **Mean** | **SD** | **All** (Intervention; usual care) |
| Community-based services | 502·8 | 501·8 | 443·4 | 396·8 | 338 (192,146) |
| Accident and Emergency | 144·4 | 238·9 | 181·9 | 350·4 | 338 (192,146) |
| Hospital care | 2,195·5 | 5,837·2 | 2,682·8 | 10,051·5 | 338 (192,146) |
| Outpatient attendances | 687·0 | 716·7 | 591·4 | 665·4 | 338 (192,146) |
| Pulmonary rehabilitation | 762·18 | 341·12 | 738·29 | 373·18 | 309 (179,130) |
| Intervention delivery and staff training | 227·21 | 110·97 | n/a | n/a | 242, n/a |

**Table 12. Mean participant total healthcare resource use cost and quality adjusted life years (QALYs)**

| | Intervention | | Usual care | | Intervention-control | | | *n* (data available) |
|---|---|---|---|---|---|---|---|---|
| | **Mean** | **SD** | **Mean** | **SD** | **Unadjusted mean difference** | **Adjusted mean difference[b]** | **95% CI** | **All** (Intervention; usual care) |
| **Total cost, £** | | | | | | | | |
| Baseline to twelve months[a] | 4401·85 | 407·36 | 5100·42 | 1164·22 | -698·56 | 770·24 | -27·91 to 1568·39 | 423 (242; 181) |
| **QALYs** | | | | | | | | |
| Baseline to 12 months[a] | 0·537 | 0·0148 | 0·549 | 0·016 | -0·011 | -0·010 | -0·042 to 0·021 | 423 (242; 181) |

**a·** Estimates from multiple imputed data;
**b**· Using multilevel mixed-effect model and controlling for baseline covariates

**Figure 2· Cost-effectiveness plane (base case results)**

Scatterplot showing the bootstrapped mean differences in health care costs and effects (QALYs) between the TANDEM intervention and control arms· Estimates based on multiple imputation for missing data·

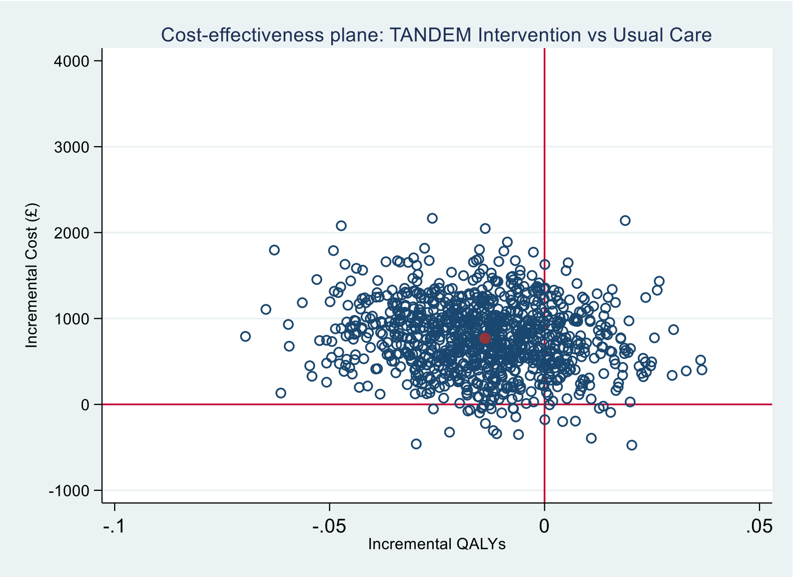

**Figure 3· Cost-effectiveness acceptability curve (base case results)**

Cost-effectiveness acceptability curve (CEAC) showing the probability that the TANDEM intervention is cost-effective compared to usual care· CEAC based on data that includes multiple imputation for missing data·

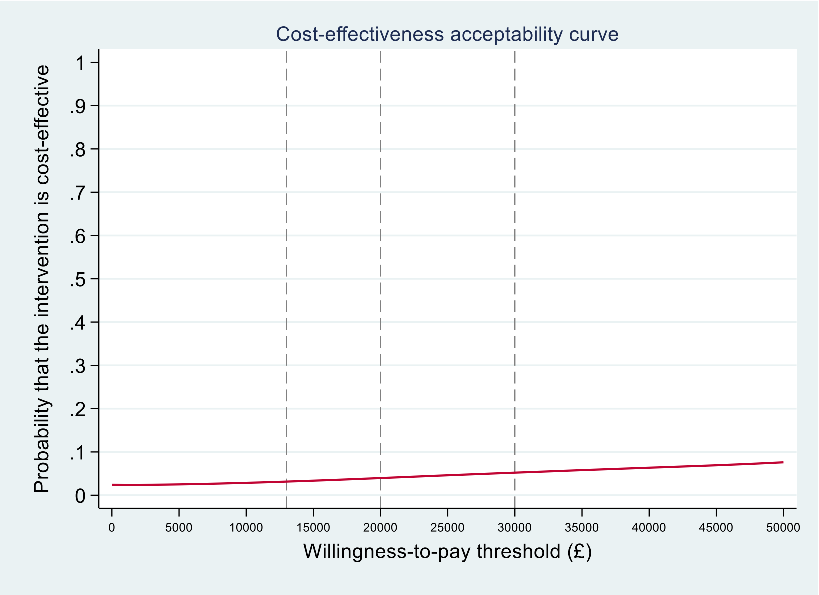


**Table 13. Estimate of incremental net health benefit (INHB) for different cost-effectiveness thresholds (base case results)**

| Willingness to pay | INHB | 95% CI | Probability cost-effective |
|---|---|---|---|
| £13000 | -0·0697 | -0·0726 to -0·0680 | 0·030 |
| £20000 | -0·0489 | -0·0512 to -0·0477 | 0·037 |
| £30000 | -0·0361 | -0·0380 to -0·0352 | 0·055 |

**Box 7. Health economics sensitivity analyses**

Two post-hoc sensitivity analyses were performed. The first repeated the base-case cost-effectiveness analysis on data 'trimmed' of one outlying control participant with an extreme cost value (arising due to a high total inpatient admission cost) and an imputed QALY value. The second considered the effect of using a more conservative estimate of the costs of training TANDEM Facilitators, based on a larger treatment caseload (5,591 cases instead of 1,288 per year) suggested by lead trial clinicians as a realistic Facilitator caseload if the intervention were implemented in a routine clinical service. Trimming the outlier from the sample increased the incremental cost of TANDEM (from £770 to £1057) and led to a zero QALY effect of TANDEM over usual care only (compared to the small negative effect on QALYs when the outlier was included). Neither trimming the outlier or using a revised estimate of training costs changed core conclusions regarding intervention cost-effectiveness or probabilities relating to decision uncertainty (Supplementary material tables 14 and 15).

**Supplementary Table 14· Sensitivity analysis: results after trimming outliers**

| Willingness to pay threshold | INB (Mean) | 95% CI | Probability cost-effective |
|---|---|---|---|
| £13000 | -0·0809 | -0·0816 to -0·0780 | 0·000 |
| £20000 | -0·0524 | -0·0529 to -0·0499 | 0·013 |
| £30000 | -0·0348 | -0·0352 to -0·0325 | 0·058 |

**Supplementary Table 15· Sensitivity analysis: results after using alternative estimation of training**

| Willingness to pay threshold | INB (Mean) | 95% CI | Probability cost-effective |
|---|---|---|---|
| £13000 | -0·0665 | -0·0689 to -0·0642 | 0·038 |
| £20000 | -0·0470 | -0·0488 to -0·0453 | 0·053 |
| £30000 | -0·0350 | -0·0364 to -0·0335 | 0·058 |

## References


| | |
|---|---|
| 1 | Puhan MA, Frey M, Büchi S, Schünemann HJ. The minimal important difference of the hospital anxiety and depression scale in patients with chronic obstructive pulmonary disease. *Health Quality of Life Outcomes* 2008; **6:** 1-6. |
| 2 | Bjelland I, Dahl AA, Haug TT, Neckelmann D. The validity of the Hospital Anxiety and Depression Scale. An updated literature review. *J Psychosom Res* 2002; **52:** 69–77. |
| 3 | Ng TP, Niti M, Tan WC, Cao Z, Ong KC, Eng P. Depressive symptoms and chronic obstructive pulmonary disease: effect on mortality, hospital readmission, symptom burden, functional status, and quality of life. *Arch Intern Med* 2007; **167:** 60–7. |
| 4 | Cameron IM, Crawford JR, Lawton K, Reid IC. Psychometric comparison of PHQ-9 and HADS for measuring depression severity in primary care. *Br J Gen Pract* 2008; **58**: 32-6. |
| 5 | Howard C, Dupont S, Haselden B, Lynch J, Wills P. The effectiveness of a group cognitive-behavioural breathlessness intervention on health status, mood and hospital admissions in elderly patients with chronic obstructive pulmonary disease. *Psychol Health Med* 2010; **15:** 371-85. |
| 6 | Livermore N, Sharpe L, McKenzie D. Prevention of panic attacks and panic disorder in COPD. *Eur Respir J* 2010; **35**: 557-63 |
| 7 | Griffiths TL, Burr ML, Campbell IA, Lewis-Jenkins V, Mullins J, Shiels K, *et al.* Results at 1 year of outpatient multidisciplinary pulmonary rehabilitation: a randomised controlled trial. *Lancet* 2000; **355:** 362–8. |
| 8 | Dowson C, Laing R, Barraclough R, Town I, Mulder R, Norris K, et al. The use of the Hospital Anxiety and Depression Scale (HADS) in patients with chronic obstructive pulmonary disease: a pilot study. *NZ Med J* 2001; **114**: 447-9 |
| 9 | Arnau RC, Meagher MW, Norris MP, Bramson R. Psychometric evaluation of the Beck Depression Inventory-II with primary care medical patients. *Health Psychol* 2001; **20**:112-9. |
| 10 | Button KS, Kounali D, Thomas L et al. Minimal clinically important difference on the Beck Depression Inventory--II according to the patient's perspective. *Psychol Med* 2015; **45**:3269-79. |
| 11 | PsychVisit. Beck Depression Inventory - 2nd Edition (BDI-II). URL: http://www.psychvisit.com/Scales/BDI-2.html (Accessed 25 October 2021) |
| 12 | Masson SC, Tejani AM. Minimum clinically important differences identified for commonly used depression rating scales. J Clin Epidemiol 2013; **7**; 805-7. |
| 13 | Beck AT, Epstein N, Brown G, Steer RA. An inventory for measuring clinical anxiety: Psychometric properties. *J Consult Clin Psychol* 1988; **56**: 893-7. |
| 14 | Kim HFS, Kunik ME, Molinari VA et al. Functional Impairment in COPD Patients : the Impact of Anxiety and Depression. *Psychosomatics* 2000; **41**: 465–71. |
| 15 | Muntingh AD, van der Feltz-Cornelis CM, van Marwijk HW, Spinhoven P, Penninx BW, van Balkom AJ. Is the Beck Anxiety Inventory a good tool to assess the severity of anxiety? A primary care study in the Netherlands Study of Depression and Anxiety (NESDA). *BMC Fam Pract*. 2011; **12:** 66. doi: 10.1186/1471-2296-12-66. |
| 16 | Jones PW, Quirk FH, Baveystock CM, Littlejohns P. A self-complete measure for chronic airflow limitation: the St George's Respiratory Questionnaire. *Am Rev Respir Dis* 1992; 145: 1321-7. |
| 17 | Jones P. St George's Respiratory Questionnaire for COPD patients (SGRQ-C). URL: https://www.sgul.ac.uk/about/our-institutes/infection-and-immunity/research-themes/research-centres/health-status/sgrq (Accessed 7 November 2021) |
| 18 | https://www.sgul.ac.uk/research/research-operations/research-administration/st-georges-respiratory- |

| | |
|---|---|
| | questionnaire/minimum-clinically-important-difference-mcid (accessed 22 Dec 2022) |
| 19 | Osborne RH, Elsworth GR, Whitfield K. The Health Education Impact Questionnaire (heiQ): an outcomes and evaluation measure for patient education and self-management interventions for people with chronic conditions. *Pat Ed Counsel* 2007; **66**:192-201. |
| 20 | Sullivan O, Gershuny J. Speed-up society? Evidence from the UK 2000 and 2015 time use diary surveys. Sociology 2018; **52**: 20-38. |
| 21 | Priebe S, Savill M, Wykes T, Bentall RP, Reininghaus U, Lauber C, et al. Effectiveness of group body psychotherapy for negative symptoms of schizophrenia: multicentre randomised controlled trial. *Br J Psychiatry* 2016; **209**: 54–61. |
| 22 | Broadbent E, Petrie KJ, Main J, Weinman J. The brief illness perception questionnaire. J *Psychosom Res* 2006; **60**: 631-7. |
| 23 | Personal Social Services Research Unit. Client Service Receipt Inventory. URL: https://www.pssru.ac.uk/csri/client-service-receipt-inventory (Accessed 19 October 2021). |
| 24 | Curtis L, Burns A. Unit Costs of Health and Social Care 2020. URL: https://www.pssru.ac.uk/project-pages/unit-costs/unit-costs-2020 (Accessed 6 November 2021). |
| 25 | National Institute for Health and Clinical Excellence. Position statement on use of the EQ-5D-5L value set for England (updated October 2019). URL: https://www.nice.org.uk/about/what-we-do/our-programmes/nice-guidance/technology-appraisal-guidance/eq-5d-5l (Accessed 6 November 2021). |
| 26 | Van Hout B, Janssen MF, Feng YS et al. Interim scoring for the EQ-5D-5L: mapping the EQ-5D-5L to EQ-5D-3L value sets. Value Health 2012; 15: 708-15. |
| 27 | Shalom JG, Aderka IM. A meta-analysis of sudden gains in psychotherapy: Outcome and moderators. Clinical Psychology Review 2020;76:101827 |
