# Supplementary material for: Tailored psychological intervention for anxiety or depression in COPD (TANDEM): a randomised controlled trial
Source: Eur Respir J. 2023 Nov 2;62(5):2300432. doi: 10.1183/13993003.00432-2023 (PMC10620475; doi:10.1183/13993003.00432-2023)
Supplement: Supplementary file 2 [file ERJ-00432-2023.Supplement_2.pdf]

Supplementary Materials 2

Tandem

Tailored intervention for ANxiety and DEpression in COPD Management

Statistical Analysis Plan

Main Trial

Version: 2.0
Date: 12/MAY/2021

Person(s) contributing to the analysis plan	
Name(s) and position(s)	Claire Chan, Statistician<br>Garima Priyadarshini, Statistician<br>Melanie Smuk, Senior statistician<br>Kim May Lee, Senior statistician<br>Richard Hooper, Senior Statistician<br>Stephanie Taylor, Chief investigator<br>Hilary Pinnock, Co-chief investigator
Authorisation	
Position	**Chief or principal investigator**
Name	**Stephanie Taylor**
Signature	Stephanie Taylor
Date	18/05/2021
Position	**Senior trial statistician**
Name	**Richard Hooper**
Signature	[illegible]
Date	18 May 2021
Position	**Independent statistician**

Name	**Prof Toby Prevost**
Tick once reviewed	✓
Date	04 May 2021

1. Administrative Information

Trial registration number: ISRCTN59537391

This Statistical Analysis Plan (SAP) is based on protocol version 8.0 (date 29/05/2019)

Statistical Analysis Plan revision history

Protocol version	Updated SAP version no.	Section number changed	List of changes from previous version/protocol	Author of change	Date
6.0	0.1	N/A	N/A	Claire Chan	12/JUL/2019
6.0	0.2	Y	Changes made in response to comments from MS	Claire Chan	06/AUG/2019
6.0	0.3	Y	Additional summaries added to section 6, consort flow diagram in appendix 2, and more tables in appendix 3	Claire Chan	12/AUG/2019
6.0	0.4	Y	Changes made in response to comments from MS	Claire Chan	16/AUG/2019
6.0	0.5	Y	Changes made in response to comments from HP, TP, and ST. In particular, primary analysis model changed from a random effects model imposing clustering in the control arm to a heteroscedastic partially nested mixed effects model which correctly accounts for the partially clustered design.	Claire Chan	20/JAN/2020
8.0	0.6	Y	Changes made in response to comments from MS	Claire Chan	03/FEB/2020

8.0	1.0	Y	Same as 0.6 (comments removed)	Claire Chan	10/FEB/2020
8.0	1.1	N	Specify method for computing p-values (it was not explicitly mentioned).<br><br>Describe sensitivity analysis to explore the impact of the pandemic	Kim May Lee	30/OCT/2020
8.0	1.2	N	Description of Satterthwaite method for computing p-values added.<br><br>Reference for Satterthwaite method added and reference numbers revised.<br><br>Changes made to sensitivity analysis for the impact of the varying allocation ratios.<br><br>Changes made to sensitivity analysis for the impact of the pandemic.<br><br>Dummy tables revised corresponding to the changes in the analyses.	Kim May Lee/ Garima Priyadarshini	08/JAN/2021
8.0	1.3	N	Additional sensitivity analysis for missing data and corresponding reference added.<br><br>Order of references revised.<br><br>Complier Average Causal Effect analysis added.<br><br>Dummy tables revised corresponding to the changes in the analyses.	Kim May Lee/ Garima Priyadarshini	25/JAN/2021
8.0	1.4	N	Dummy tables added for baseline comparisons.	Garima Priyadarshini	16/MAR/2021
8.0	1.5	N	Reference for CACE added and reference list rearranged.	Richard Hooper/ Garima Priyadarshini	19/MAR/2021

			Dates added for randomisation periods.<br><br>Dummy tables modified.		
8.0	1.6	N	Revisions made in response to comments from TP	Richard Hooper/ Garima Priyadarshini	23/APR/2021
8.0	1.7	N	Details of randomisation periods in Section 5.7 were incorrect in v1.6: the intention was to divide into periods within which allocation ratio appeared constant (1.25:1 in period 1; 5:1 in period 2; 1:1 in period 3).	Richard Hooper/ Garima Priyadarshini	18/MAY/2021
8.0	2.0	N	Same as 1.7 (comments removed)	Richard Hooper/ Garima Priyadarshini	18/MAY/2021

Members of the writing committee

Claire Chan, Queen Mary University of London (QMUL), wrote the statistical analysis plan, with input from Melanie Smuk, QMUL, Stephanie Taylor, QMUL, and Hilary Pinnock, University of Edinburgh. Kim May Lee, QMUL, Garima Priyadarshini, QMUL, and Richard Hooper, QMUL, updated the SAP to version 2.

Timing of Statistical Analysis Plan revisions in relation to unblinding of data/results

All inputting members were blind to the data until the statistical analysis plan was signed off. The members were present at open DMC meetings only, and closed DMC reports were prepared by a statistician independent of the trial team.

Remit of Statistical Analysis Plan

We provide here details of the statistical analyses and tables to be reported within the principal paper(s) of the Tandem trial. Any exploratory, post hoc or unplanned analyses will be clearly identified in the report. Subsequent papers of a more exploratory nature will not be bound by this strategy but will be expected to follow the broad principles laid down in it. The remit of this statistical analysis plan covers the quantitative aspect of objectives 1 and 3 of the main trial. Health Economic outcomes and process outcomes are addressed in the health economic analysis plan and process evaluation analysis plan respectively.

Abbreviations

BAI	Becks Anxiety Inventory
BDI-II	Becks Depression Inventory
B-IPQ	Brief Illness Perception Questionnaire

BLF	British Lung Foundation
CBA	Cognitive Behavioural Approach
COPD	Chronic Obstructive Pulmonary Disease
FEV_1/FVC	Forced Expiratory Volume in 1s/Forced Vital Capacity
HADS	Hospital Anxiety and Depression Scale
HeiQ	Health Education Impact Questionnaire
ICC	Intraclass correlation coefficient
ITT	Intention-to-treat
MAR	Missing At Random
MCAR	Missing Completely At Random
mMRC	Modified Medical Research Council
MNAR	Missing Not At Random
PR	Pulmonary Rehabilitation
SAP	Statistical Analysis Plan
SGRQ	St George's Respiratory Questionnaire
WEMWBS	Warwick-Edinburgh mental Wellbeing Scale
ZBI	Zarit Burden Interview

2. Background and trial design

Give a brief summary of the trial. This should include the points listed in the table below.

**Study objectives**	Study aim<br>To refine, pilot and evaluate a tailored, psychological cognitive behavioural approach (CBA) intervention, which links into, and optimises the benefits of routine pulmonary rehabilitation, with the aim of reducing mild/moderate anxiety and/or depression in people with moderate, severe or very severe COPD.<br><br>*Phase I – Pre-pilot study –* completed (Refinement of the TANDEM CBA intervention manual as a result of focus groups/individual interviews with patients, carers, and health care professionals)<br><br>*Phase II (Internal pilot and feasibility study) – completed (Separate SAP: Statistical analysis plan v1.0 Tandem internal pilot)*<br><br>*Phase III (Main Trial) – Aim & objectives*<br>To undertake a randomised controlled trial (and a process evaluation) to:

	1) Examine the clinical effectiveness of the CBA intervention on clinical outcomes compared to usual care (the offer of pulmonary rehabilitation (PR) alone).<br>2) Examine the process outcomes.<br>3) Examine the effect of the CBA intervention on carers (where appropriate).<br>4) Determine the cost effectiveness of the CBA intervention from an NHS and personal social services perspective.<br>*5)* Conduct a process evaluation to inform the implementation of the CBA intervention if the trial is positive, or assist interpretation of findings if it is negative.
**Study design**	Multi centre, two-arm, parallel group, pragmatic, individually randomised controlled, superiority trial including an internal pilot. The designed randomisation ratio is 1.25:1 in favour of the intervention.
**Setting**	Participants will be recruited from primary and secondary care, and from PR services. The intervention will be delivered in participant's own homes, or at a convenient local (usually NHS) facility, and over the phone.
**Participants**	**<u>Inclusion Criteria – Participants</u>**<br>• Adults with a confirmed diagnosis of COPD, post bronchodilator FEV_1/FVC ratio <70%.<br>• Moderate, severe or very severe COPD severity on spirometry, FEV_1 <80% predicted.<br>• Participants with probable mild or moderate anxiety as determined by the Hospital Anxiety and Depression Scale Anxiety Subscale (HADS-A) scores ≥8 to ≤15; and/or probable mild or moderate depression as determined by Hospital Anxiety and Depression Scale – Depression Subscale (HADS-D) scores ≥8 to ≤15.<br>• Eligible to attend assessment appointment at their local pulmonary rehabilitation service at the time of randomisation i.e. 12 months have elapsed since last undertook PR or participant has another indication for PR referral (e.g. recent deterioration; recent hospitalisation with an acute exacerbation of COPD).<br>• Participants who have been offered PR previously but declined the offer or did not complete PR will be included.<br><br>**<u>Exclusion Criteria – Participants</u>**<br>• Participants with both HADS-A score and a HADS-D score <8 (within normal range).<br>• Unable to give valid consent.<br>• HADS-D or HADS-A score greater than 15 (suggestive of possible severe anxiety/depression).<br>• If a referral to PR has been made, the participant is ineligible if they have agreed to attend PR sessions and are due to commence < 4 weeks after the screening visit, (as confirmed in a PR appointment letter or by the PR team). The rationale for this exclusion is that the participant will not have time to receive a sufficient "dose" of the intervention prior to starting PR.

	• Severe uncontrolled psychological or psychiatric disorder e.g. schizophrenia, bipolar disorder, struggling to cope as a result of their personality disorder, uncontrolled substance abuse (alcoholism, illegal drugs) that would make them unsuitable for the intervention.<br>• Ineligible for pulmonary rehabilitation at their local PR service at the time of randomisation (typically if they had undertaken a course of PR in the last 12 months and there were no new clinical indications for PR[20].<br>• A co-morbidity so severe it would prevent the participant from engaging fully in the intervention/ control e.g. rheumatoid arthritis or osteoarthritis, multiple sclerosis, chronic widespread pain syndrome, previous cerebral vascular event such that the participant would not be eligible for pulmonary rehabilitation.<br>• Participants with moderate/severe cognitive impairment.<br>• In receipt of a psychological intervention primarily directed at helping to manage anxiety or depression in the last 6 months (NB those on antidepressants/ anxiolytics not excluded) or in receipt of a referral for a psychological treatment (e.g. CBT therapy) for management of anxiety or depression. (NB those in receipt of counselling for bereavement or smoking cessation are not excluded).<br>• Participants currently involved in another clinical trial related to COPD (to reduce study participation burden on participants).<br>• Not sufficiently fluent in English to be able to complete the intervention, questionnaires (NB the questionnaires are supervised self-complete, but can be read to participants if necessary, so poor literacy would not exclude individuals who are otherwise sufficiently fluent in English).<br><br>**Inclusion Criteria – Carers**<br>• Identified by a participant with COPD in the study as a 'particular family caregiver or friend who helps them' and whom they would be happy for us to invite to join the study.<br><br>**Exclusion Criteria – Carers**<br>• Unable to give valid consent.<br>• Not sufficiently fluent in English to be able to complete the questionnaires.
**Interventions**	The designed randomisation ratio is 1.25:1 in favour of the intervention (usual care plus a tailored psychological intervention) compared to the control (usual care).<br><br>**Intervention**<br>The TANDEM intervention to optimise the potential synergy between the psychological one-to-one CBA intervention and PR. The CBA intervention will precede PR and target individuals' cognitions and behaviours associated with anxiety and depression to decrease psychological morbidity and increase self-efficacy (confidence) and motivation among participants with moderate to very severe COPD to attend and complete PR.<br><br>**Control**<br>Usual care will follow local arrangements for provision of PR to people with COPD referred to the service, and participants will attend the usual multidisciplinary PR course (including any psychological treatment provided routinely in that service).

	In agreement with the local service (who may prefer to use their own materials), we will provide the British Lung Foundation (BLF) DVD on living with COPD, and booklets on COPD and pulmonary rehabilitation. Participants will also be eligible for referral to other services, e.g. referral for Improving Access to Psychological Therapies (IAPT) services, at the discretion of their usual healthcare providers.
**Primary outcome measure(s)**	Co-primary primary outcomes:<br>Depression and Anxiety (HADS-D and HADS-A) at 6 months

3. Outcome measures

Primary outcome

The co-primary outcomes are participant depression and anxiety (HADS-D and HADS-A) at 6 months

Secondary outcomes

- Participant Depression (HADS-D) at 12 months
- Participant Anxiety (HADS-A) at 12 months
- Participant Depression (BDI-II) at 6 and 12 months
- Participant Anxiety (BAI) at 6 and 12 months
- Participant Smoking status at 6 and 12 months
- Participant Respiratory Health-related quality of life (SGRQ) at 6 and 12 months
- Participant Illness perceptions about COPD (B-IPQ) at 6 and 12 months
- Participant Social engagement (heiQ) at 6 and 12 months
- Participant Social functioning (Time Use Survey) at 6 and 12 months
- Carer Burden Interview (ZBI) at 6 and 12 months
- Carer Mental Well-being (WEMWBS) at 6 and 12 months

See Appendix 1 for information on timing of data collection, and see Appendix 2 for information on how outcomes are derived.

Other secondary outcomes

Health Economic outcomes (Participant Quality of life (EQ-5D-5L), Client Service Receipt Inventory, and Health Care Resource Use) are addressed in the health economic analysis plan.

Process outcomes are addressed in the process evaluation analysis plan.

4. Sample size and randomisation

Sample size calculation
Intervention (usual care plus a tailored psychological intervention): 240; Control (usual care): 190

Sample size calculations are based on two primary outcomes (HADS-A, anxiety, subscale at 6 months, and HADS-D, depression, subscale at 6 months). Based on a two-sided significance level of 2.5% and 90% power, recruiting 153 participants would allow us to detect a difference of 1.7 points on the HADS anxiety subscale, and 1.5 points on the HADS depression subscale (based on a standard deviation (SD) of 4.2 for anxiety and 3.6 for depression [1]): these are equivalent to a standardised mean difference of about 0.4, and are similar to the minimum clinically important difference of 1.5 for HADS in COPD.[2] Due to the clustering effect by therapist in the intervention arm, we increased the sample size. Assuming an intraclass correlation coefficient (ICC) between therapists of 0.01 and 24 participants per therapist leads to a design effect of 1.23, which required increasing the number of participants in the intervention arm to 189 (342 overall). Assuming a dropout rate of 20%, we would require 428 participants overall. This has been rounded up to 430. Using an allocation ratio of 1.25 vs. 1, this would lead to approximately 240 participants in the intervention arm and 190 in the control arm. We have chosen this unbalanced allocation ratio as it will maximise power compared to a 1:1 ratio due to the presence of the anticipated size of the ICC resulting from clustering by therapist in the intervention arm only.

Randomisation procedure

Participants will be randomised to either the intervention or usual care. The allocation ratio will be 1.25:1 ratio in favour of the intervention. Randomisation will be performed by stratification by NHS Trust, and using minimisation within each stratum with a random element of 0.80,[3] balanced for important participant characteristics to ensure treatment groups are well matched at baseline. Stratifying by NHS Trust promotes a comparable distribution of participants across NHS Trusts within the two treatment groups, which is important because although extensive discussion with current applicants, experts and services themselves suggested that PR services should not vary too much within Trusts, they could vary *between* trusts. Randomisation will be implemented using a central online randomisation service based at Queen Mary University of London. Allocation concealment will be maintained through the use of the centralised service.

The NHS Trusts are as follows, although more Trusts may be added in the future:

1) Imperial College Healthcare NHS Trust
2) Homerton University Hospital NHS Foundation Trust
3) University Hospitals of Leicester NHS Trust
4) Leicestershire Partnership NHS Trust
5) Guy's and St Thomas' NHS Foundation Trust
6) University Hospitals Coventry and Warwickshire NHS Trust
7) South Warwickshire NHS Foundation Trust
8) Sandwell and West Birmingham Hospitals NHS Trust
9) King’s College Hospital NHS Foundation Trust

10) Berkshire Healthcare NHS Foundation Trust
11) Southern Health NHS Foundation Trust
12) North Bristol NHS Trust

The minimisation factors are:

1) HADS-Anxiety Scale, with categories: 0-7, 8-10, 11-15
2) HADS-Depression Scale, with categories: 0-7, 8-10, 11-15
3) Modified MRC Breathlessness Scale, with categories: 0-2, 3-4
4) Self-reported smoking status, with categories: smoker, non-smoker (ex-smoker or never smoked)

5. Analysis methods

5.1 Baseline characteristics

Baseline characteristics and questionnaires will be summarised for each treatment group by the mean and standard deviation (SD) or median and interquartile range for continuous variables, and the number and percentage for categorical variables. See Table 1,
Table 2, Table 3, and Table 4 in Appendix 4.

5.2 Adherence to treatment

For our analysis, a participant in the intervention arm is considered to have completed CBA (typically 6-8 sessions) if they complete 2 or more sessions (as per protocol, completion of two sessions considered minimum effective dose of the intervention). A participant in either arm of the study is considered to have completed PR if they complete 75% or more of their scheduled PR sessions. See Table 97.

5.3 Information for CONSORT flow diagram

See Appendix 3 for a dummy CONSORT flow diagram.

5.4 General analysis principles

Analyses will follow the intention-to-treat (ITT) principle. This requires that all participants be included in the analysis according to the treatment group to which they were randomised, regardless of any departures from randomised treatment.[4] Anyone identified post-randomisation as having been ineligible for the trial at the time they were randomised is not considered a 'participant' in this sense, and will be excluded from all analyses (a count of these will be provided in the CONSORT flow-chart). Where it is not possible to follow up participants, we will handle missing data by including all those with a recorded outcome.[5] A secondary analysis will estimate the complier-average causal effect (CACE), based on adherence as defined above. This is the intervention effect in participants who would adhere to the intervention, and is estimated in a way that respects randomisation (in this regard it is superior to a per protocol analysis).[6]

<u>Continuous outcomes</u>

Analyses will be presented as:

- The number of participants included in the analysis, by treatment group;
- A summary measure of the outcome, by treatment group (e.g. mean (SD);
- A treatment effect (difference in means) with a 95% confidence interval;
- A two-sided p-value

For all analyses, a significance level of 5% will be used, except when the Hochberg procedure is applied to account for there being two primary outcomes.

All analyses will account for the partially clustered design of the study, due to the clustering effect by facilitator in the intervention arm only. Each participant in the intervention arm will be defined as belonging to a cluster, defined by which facilitator they belonged to. It is possible that the facilitator could change for a participant, for example if a facilitator withdraws and a new facilitator is assigned to a participant. In such scenarios, for analysis purposes we will consider that the participant is clustered within the facilitator for which they have had the majority of their sessions. If they had equal amounts of sessions under multiple facilitators, we will take the first of such facilitators. The clustering effect by facilitator in the intervention arm will be modelled using a partially nested mixed-effects model, which confines the random effect for cluster to the intervention arm only. If we write out the model for the two levels of treatment, this essentially amounts to a random intercept for each cluster in the intervention arm and one intercept for the unclustered control arm.[7, 8] In order to run the model in Stata, we treat each participant in the control arm as a single cluster, and allow the residual variance to be different in the intervention and control arms.[7]

All analyses will also account for the correlation between outcomes at 6 and 12 months using a random effect for participant. This approach will provide unbiased estimates even if some participants only provide data at one of the two time points, under the missing at random assumption implied by the model. We fit a heteroscedastic model, allowing for different residual level errors in the two treatment arms since we might expect participants in the intervention arm to vary in a different way to those in the control arm.[7, 8] The model will be estimated using restricted maximum likelihood (REML). Treatment arm, time point (month 6 or 12), and the interaction between treatment arm and time point will be included in the model as fixed factors.

All analyses will adjust for the outcome measured at baseline whenever possible. Moreover, analyses will reflect the design of the study, and so stratification and minimisation variables will be adjusted for.[9] These covariates will not be interacted with time point. Continuous covariates will be fitted for the HADS-A and HADS-D screening scores. Although categories were used for HADS-A and HADS-D during the minimisation, we avoid unnecessary categorisation of the covariates for the analysis as this should increase power.[10] Continuous covariates will be assumed to have a linear relationship with the outcome. Binary covariates will be fitted for the baseline degree of breathlessness (categories: 0-2 and 3-4) and baseline smoking status (categories: smoker and non-smoker (ex-smoker/never smoked)). We also adjust for NHS Trust as a fixed categorical variable. Due to the large number of NHS Trusts, one could fit a random effect for Trust, but this would result in a complex (four-level) mixed effect model with concerns about fitting due to the sample size and so in this situation a fixed effect is preferable. Moreover, the other stratifiers are nested within Trust. Furthermore, when there are a large number of participants per centre (25 or more, which we expect here), random and fixed effect perform equally well in terms of coverage, power and efficiency.[11, 12]

See Table 7 for how we will present the main results for the primary and secondary continuous outcomes.

Binary outcomes

The only binary secondary outcome is smoking status at 6 and 12 months (~~current~~ smoker vs non-smoker). Decreased smoking cessation in the intervention arm is psychologically plausible with the Tandem intervention (increased self-efficacy around condition management increasing likelihood of attempting and succeeding in quitting). We will attempt to follow an analysis strategy similar to that outlined above for continuous outcomes, but using mixed logistic regression in place of mixed linear regression, and introducing any simplifications as required to produce an effect estimate if there are problems with model convergence (see strategy in next section). There may not be a great deal of movement in this outcome from baseline making it more of a challenge to analyse than other outcomes. It is also of less direct interest than other secondary outcomes, and a simpler analysis may be an acceptable trade-off.

See Table 8 for how we will present the results.

Other analyses involving binary outcomes, such as CBA intervention attendance and completion rates, and PR attendance and completion rates, are discussed in Section 6.

Model convergence issues
If a model fails to converge, the following strategy will be employed, with changes considered in the order presented:

1	Remove the correction allowing for a small number of clusters (Satterthwaite correction – see model specification below)
2	Remove the minimisation covariates
3	Remove the baseline assessment of outcome as a covariate
4	Split the two time-points into two separate models – i.e. remove the fixed effect for time and the random effect for participant
5	Fit a homoscedastic model – i.e. one overall residual variance (independent residual structure) rather than separate variances estimated for the two treatment groups (could be one of two alternatives – either assuming the control arm is a single 'cluster', or that each individual in the control arm is a separate 'cluster')
6	Remove the random effect for facilitator

Missing data for baseline covariates
We do not expect missing data for any of the baseline covariates for the primary analysis. It is possible that there will be a small amount of missing data for the secondary outcomes measured at baseline. In this case, missing data for baseline covariates to be included in the analysis model will be accounted for using mean imputation.[13]

Missing data for outcomes
For outcomes that are measured at multiple time points during follow-up, we have based our analysis strategy on that proposed by White et al 2011.[5] To deal with incomplete data (i.e. when participants have missing data at one of the follow-up time points) we will:

1. Attempt to follow up all randomised participants even if they withdraw from the allocated treatment (but remain in the study)
2. Perform a main analysis of all observed data that are valid under a plausible assumption about the missing data
3. Perform sensitivity analyses to explore the effect of departures from the assumptions made in the main analysis
4. Account for all randomised participants, at least in the sensitivity analyses

For the main analyses (point 2), we will include all participants with at least one post-randomisation assessment (i.e. if the relevant outcome is recorded for at least one follow-up time point) in the analysis. Participants with missing outcome data at both 6 and 12 months will be excluded for this analysis. The mixed-effects model adjusted for baseline covariates assumes that the data are Missing at Random (MAR).[14] Modelling of the observed data in this way is a principled method to deal with missingness, as information is 'borrowed' from other clusters. This strategy of analysis has been widely recommended in the presence of missing outcome data. For outcomes that consist of several items combined to create a score, we expect most participants will either complete all or none of the items. Only participants who completed all of the questions which form the score at **either** 6 or 12 months will be included in the primary analysis.

We perform sensitivity analyses for the primary outcomes to assess the robustness of our primary analysis to the missing data assumptions (point 3) and account for all the participants analysed, including those lost to follow up or withdrawn (point 4). See section 5.6.

5.5 Analysis of co-primary outcomes

We will analyse the co-primary outcomes separately. This is equivalent to fitting a joint model in the case of no missing data. A joint model would be more efficient in the case where one outcome is missing but the other outcome is not. However, a joint model is more computationally complex and it is unlikely that participants would complete the HADS-A questions but not the HADS-D, and vice versa.

A heteroscedastic partially nested mixed-effects model will be fitted for HADS-A and HADS-D, as follows:

$$\text{Outcome} = \beta_0 + \beta_1\text{time} + \beta_2\text{treat6} + \beta_3\text{treat12} + \beta_4\text{trust1} + \ldots + \beta_{14}\text{trust12} + \beta_{15}\text{hadsa} + \beta_{16}\text{hadsd} + \beta_{17}\text{breath} + \beta_{18}\text{smoke} + u_1 + \varepsilon$$

where:

time	= Measurement occasion	= 0 if 6 months; 1 if 12 months
treat6	= Effect of treatment at 6 mo	= 1 if intervention arm and 6 months; 0 otherwise
treat12	= Effect of treatment at 12 mo	= 1 if intervention arm and 12 months; 0 otherwise
trust	= NHS Trust	= 0 if trust 1; 1 if trust 2; …; 11 if trust12 (assuming 12 trusts)
hadsa	= Baseline HADS-A	= (continuous)
hadsd	= Baseline HADS-D	= (continuous)
breath	= Baseline breathlessness	= 0 if "0-2"; 1 if "3-4"
smoke	= Baseline smoking status	= 0 if non-smoker; 1 if smoker
u_1	= Participant random effect	
ε	= Residual error	

u_2|covariates ~ $N(0, \sigma^2_{u2})$
u_1|covariates, u_2 ~ $N(0, \sigma^2_{u1})$
ε|(treat=0) ~ $N(0, \sigma^2_{\varepsilon 0})$
ε|(treat=1) ~ $N(0, \sigma^2_{\varepsilon 1})$

The analysis will be implemented in Stata as follows:

```
mixed outcome time treat6 treat12 i.trust hadsa hadsd smoke breath ///
        || clusterid: ///
        || participantid:, ///
        stddev reml dfmethod(sat) residuals(independent, by(treat))
```

where the clusterid represents the facilitator for each participant in the intervention arm and each individual participant in the control arm (see section 5.4).

With mixed regression modelling there is a risk that the type I error rate will be inflated when the number of clusters is small, and a variety of corrections have been proposed. We will use the Satterthwaite method (the 'dfmethod(sat)' option in Stata), as recommended by Candlish et al.[8,16,17]

We will use a Hochberg procedure to analyse the two primary outcomes.[15] The Hochberg procedure states that if either outcome has a p-value <0.025 then that outcome is statistically significant; additionally, both outcomes are significant if both p-values are <0.05.

5.6 Complier Average Causal Effect (CACE) analysis for co-primary outcomes

As a secondary analysis we will estimate the Complier Average Causal Effect (CACE) for each co-primary outcome, with compliance defined as attending at least two CBA sessions. The CACE analysis will be performed with a latent class modelling approach, using the *gllamm* command in Stata (Generalised Linear Latent & Mixed Models; http://www.gllamm.org/books/cace.html). This will assume the same mixed model as in the intention-to-treat analysis, above, with repeated assessments at 6 and 12 months, clustering in one arm and heteroscedasticity between the arms.

5.7 Sensitivity analyses for co-primary outcomes

Missing data
For the primary analysis, we do not expect missing data for any of the baseline covariates because they are all randomisation variables. However, there may be some missing outcome data. In the primary analysis, participants who complete all of the questions that form the score at either 6 or 12 months are included; participants with missing outcome data at both 6 and 12 months are excluded. This mixed-effects model adjusted for baseline covariates assumes the data are MAR.

We will perform the following sensitivity analyses to assess the robustness of our primary analysis to different assumptions regarding the missing data. These sensitivity analyses will be performed for the co-primary outcomes (HADS-A and HADS-D at 6 months):

- A complete case analysis which assumes data missing at six months is missing completely at random (MCAR)
- An analysis in which participants with partially incomplete HADS-A or HADS-D have missing items imputed
- An analysis which assumes that data missing at six months is missing not at random (MNAR)

For the complete case analysis, we will fit the primary analysis model but only include participants with fully recorded data at six months. Participants who did not complete all components of the HADS-A or HADS-D questions respectively at six months will be excluded from the corresponding analysis.

For the second analysis we will follow the 'half-rule' suggested by Bell and colleagues, in which missing HADS items are imputed using the relevant subscale mean, as long as at least half the items in the subscale are non-missing.[18] For the third analysis, we will assess the primary outcomes under a range of MNAR scenarios. This will be done following the simple approach proposed by White et al 2007.[19] We use the formula $\Delta = \Delta_{CC} + Y_1P_1 - Y_2P_2$, where Δ is the treatment effect under the MNAR scenario, Δ_{CC} is the treatment effect from the complete case analysis on six month data above, Y_1 and Y_2 are the assumed six month mean responses for participants with missing data in treatment groups 1 and 2 respectively, P_1 and P_2 are the proportion of participants who were excluded from the six month analysis in groups 1 and 2 respectively, and groups 1 and 2 represent the intervention and control groups respectively. The standard error for Δ is assumed to be approximately equal to the standard error for $\Delta_{CC.}$ Y_2 will be varied for both outcomes between -10, -5, -1.5, 0, 1.5, 5, and 10. Negative values indicate the participant got less anxious/depressed at 6 months, positive values indicate they got more so, and a value of 0 indicates there was no change from baseline. For each value of Y_2, Y_1 will be set to Y_2 - 5, Y_2, and Y_2 + 5. For example, for Y_2 = 10 and the HADS-A outcome, this would indicate an assumption that participants in treatment arm 2 (the control arm) who were lost to follow-up at 6 months, had gained 10 points on the HADS-A subscale on average at 6 months. Y_1 would vary between 5, 10, and 15, indicating the assumption that participants in treatment arm 1 (the intervention arm) who were lost to follow-up had gained 5 points on the HADS-A subscale on average at 6 months (5 points less than those in the control arm), 10 points (the same amount as those in the control arm), or 15 points (5 points more than those in the control arm). It is possible that the value of Δ that we obtain may be implausible if it goes outside the score range of 0-21. In such cases we will truncate Δ by the scale boundary. We will note any "tipping point" – the value the treatment effect in the non-responders would need to be to change conclusions.

Finally, we will compare the distribution of baseline characteristics of people included in the primary analysis model and those missing the primary outcome at both 6 and 12 months.

Moreover, we will present the characteristics of people who have missing follow-up data because they died.

Inclusion criteria

We will perform a sensitivity analysis to assess the impact of including participants in the analysis who have less room for improvement during follow-up. The inclusion criteria state that participants must have a score of 8 or more on either the HADS anxiety or depression subscale (but not necessarily both), so some participants may have a score of <8 on one of these subscales and therefore have less room for improvement

during follow-up. We will assess the impact of including such participants, by repeating the primary analyses for HADS-A and HADS-D but excluding participants who have a score of <8 on the HADS-A and HADS-D subscales respectively. The anxiety and depression subscales of HADS are strongly associated,[20] meaning that it is unlikely that many participants have scores a lot lower than 8 on either subscale.

Time to pulmonary rehabilitation
We will assess the impact of any difference in time from baseline to attending PR in the intervention and control groups. We will summarise the time to PR in the two groups, presenting the mean (standard deviation) and the median (interquartile range). As a sensitivity analysis to the analysis of co-primary outcomes described above we will repeat the primary analysis model but with time to PR as an additional covariate.

Internal pilot
We will perform a sensitivity analysis excluding the internal pilot participants from the primary analysis, to assess the potential impact of a 'learning effect' in the pilot. For example, it is possible that the delay between completion of training and the start of intervention delivery in the internal pilot may have resulted in the treatment looking less effective in the pilot than later participants.

Allocation ratio
We know that there was a problem with the randomisation system leading to the allocation ratio being different in different periods of the trial. Specifically, over the first 69 randomisations (randomisation period 1) the observed allocation ratio was around 1.25:1 as expected; over the next 70 randomisations (randomisation period 2) the observed allocation ratio was around 5:1; this deviation from the expected allocation ratio led to a Corrective Action and Preventive Action plan that included migrating the randomisation system to a new platform, and for the remainder of randomisations (randomisation period 3) the observed allocation ratio was around 1:1 as was specified on the new platform in order to return the overall allocation ratio to a figure closer to 1.25:1. We will fit the same primary analysis model as specified in Section 5.5 but with randomisation period as an additional covariate. We will also look at the measured characteristics of participants to see if there is evidence that they are different in each time period, although Tandem is a fairly rapidly recruiting trial and we do not expect this to be the case. We will also report the balance of minimisation factors in each NHS Trust – see Table 5.

Covid 19
In late March 2020 the UK government imposed a "stay at home" order in light of the spread of COVID-19. Recruitment was due to be stopped on 30 March 2020 but curtailed about 11 days earlier due to COVID-19. Nevertheless, 427 out of 430 (target) patients were successfully recruited to the study.

We will perform a sensitivity analysis to assess the impact of the COVID-19 pandemic on the results of the co-primary outcomes. This assessment will be undertaken by splitting data in the following two ways:

1. whether data were collected before or after 19 March 2020, which is the assumed cut-off date for pre-pandemic and during pandemic outcomes.
2. whether the mode of intervention delivery was i) fully face-to-face or, ii) partially or fully via remote sessions.

The aim of this two-fold splitting is to explore several mechanisms through which COVID 19 may have impacted the trial outcomes.

For each way of splitting the data we will perform two analyses:

a) a sensitivity analysis repeating the primary analysis model but with trial period as an additional covariate (i.e. an indicator variable that specifies for each outcome assessment in the analysis whether the period should count as being before or after the cut-off);
b) an analysis to see whether the effect of the intervention is modified by trial period, performed by adding an interaction between the treatment effect at 6 months and trial period, and an interaction between the treatment effect at 12 months and trial period, to the analysis model in (a).

We will also look at measured baseline characteristics of participants to examine any evidence for differences between trial periods.

See Table , Table , Table , and Table for how we will present the results of the sensitivity analyses for the co-primary outcomes.

5.8 Subgroup analyses

None

5.9 Analysis of secondary outcomes

Participant Depression (HADS-D) at 12 months
This outcome is included in the same model as the HADS-D primary outcome.

Participant Anxiety (HADS-A) at 12 months
This outcome is included in the same model as the HADS-A primary outcome.

Participant Depression (BDI-II) at 6 and 12 months
These outcomes will be analysed using the same model as the primary outcomes at 6 and 12 months, with baseline BDI-II as an additional covariate in the model.

Participant Anxiety (BAI) at 6 and 12 months
These outcomes will be analysed using the same model as the primary outcomes at 6 and 12 months, with baseline BAI as an additional covariate in the model.

Participant Smoking status at 6 and 12 months
We will attempt to analyse smoking status using a mixed logistic regression model similar to the mixed linear regression model for the primary outcomes, though we anticipate a greater risk of model convergence issues in this case and some simplification to the analysis strategy may be required. See Table 8.

Participant Respiratory Health-related quality of life (SGRQ) at 6 and 12 months
Separate models will be fitted for the three component scores (symptoms, activity, impact) and the total score. These outcomes will be analysed using the same model as the primary outcomes at 6 and 12 months, with the relevant component score or total score for SGRQ at baseline as an additional covariate in the model.

Participant Illness perceptions about COPD (B-IPQ) at 6 and 12 months
There are 8 items, with each item scored from 0-10. Each item of the Brief IPQ assesses one dimension of illness perceptions (see Appendix 2). Separate models will be fitted for the 8 scores. These outcomes will be analysed using the same model as the primary outcomes at 6 and 12 months, with the relevant component score for B-IPQ at baseline as an additional covariate in the model.

Participant Social engagement (heiQ) at 6 and 12 months
These outcomes will be analysed using the same model as the primary outcomes at 6 and 12 months, with baseline heiQ as an additional covariate in the model.

Participant Social functioning (Time Use Survey) at 6 and 12 months
We will analyse the time (minutes) spent doing activities over the last 4 days. This outcome will be analysed using the same model as the primary outcomes at 6 and 12 months, with baseline time (minutes) spent doing activities over the last 4 days as an additional covariate in the model. Time spent doing activities over the last 4 days will be assumed to have a linear relationship with the outcome.

Carer Burden Interview (ZBI) at 6 and 12 months
These outcomes will be analysed using the same model as the primary outcomes at 6 and 12 months, with baseline ZBI as an additional covariate in the model.

Carer Mental Well-being (WEMWBS) at 6 and 12 months
These outcomes will be analysed using the same model as the primary outcomes at 6 and 12 months, with baseline WEMWBS as an additional covariate in the model.

5.10 Interim analyses

There are no formal interim analyses planned. The Data Monitoring Committee will see unblinded interim data summaries in closed session. These closed reports are produced by a statistician independent of the study team.

5.11 Blinding of the statistical analysis

Analysis cannot be blinded because of the allocation ratio. As far as possible all cleaning and checking of the data will be done before the statistician has access to the allocation codes.

5.12 Analysis software

The analysis will be carried out using Stata version 14 or newer. R version 3.6.1 or newer may be used if necessary. The software and version number used will be referenced with any analysis write up.

6. Other analyses, data summaries, and graphs

Data completeness

We will report on completeness of data on questionnaires. See Table 6.

Compliance with the intervention

We will report on CBA intervention attendance and completion rates, and PR attendance and completion rates. See Table 97 – CBA and PR attendance and completion.

Section 5.2 outlines our definitions of completion of CBA and PR. We will calculate the rates as follows:

CBA intervention attendance rate = o/p, where

o = number who attended/received at least one CBA session
p = number allocated to intervention

CBA intervention completion rate = q/o, where

q = number who completed CBA
o = number who attended/received at least one CBA session

The number who attended at least one CBA session, o, can be obtained from the number of patients with a session number for the "SESSNO" variable for at least one session (and without an entry for "FAILDELIV" - failure to deliver). The number who completed CBA, q, can be obtained from the number of patients who attended at least two CBA sessions. Both these variables are recorded in the 'CBA Facilitator – CBA session contact log' section of the database. The number allocated to the intervention, p, can be obtained from the randomisation database.

PR intervention attendance rate = r/s, where

r = number who attended/received at least one PR session
s = number referred and deemed suitable to attend PR

PR intervention completion rate = t/r, where

t = number who completed PR
r = number who attended/received at least one PR session

The number referred to attend PR, s, can be obtained from the number of patients with a value of one or more for the "NUMBER OF PR SESSIONS SCHEDULED " variable. The number who attended at least one PR session, r, can be obtained from the number of patients with a value of one or more for the "NUMBER OF PR SESSIONS COMPLETED " variable. The number who completed PR, t, can be obtained from those with a value of 0.75 or more for the ratio of "COMPLPR" to "SCHEDPR". All these variables are recorded in the 'collection of PR attendance and completion data' section of the database.

Safety analyses

We will report on adverse events and serious adverse events. See Table 19 and Table

7. References

1. Gudmundsson, G., et al., *Risk factors for rehospitalisation in COPD: role of health status, anxiety and depression.* Eur Respir J, 2005. **26**(3): p. 414-9.
2. Puhan, M.A., et al., *The minimal important difference of the hospital anxiety and depression scale in patients with chronic obstructive pulmonary disease.* Health and quality of life outcomes, 2008. **6**(1): p. 46.
3. Brown, S., et al., *Minimization—reducing predictability for multi-centre trials whilst retaining balance within centre.* Statistics in medicine, 2005. **24**(24): p. 3715-3727.
4. Moher, D., et al., *CONSORT 2010 explanation and elaboration: updated guidelines for reporting parallel group randomised trials.* Journal of clinical epidemiology, 2010. **63**(8): p. e1-e37.
5. White, I.R., et al., *Strategy for intention to treat analysis in randomised trials with missing outcome data.* BMJ, 2011. **342**: p. d40.
6. Hewitt, C.E., Torgerson, D.J. and Miles, J.N., *Is there another way to take account of noncompliance in randomized controlled trials?*. Cmaj, 2006. **175**(4), p.347-347.
7. Flight, L., et al., *Recommendations for the analysis of individually randomised controlled trials with clustering in one arm – a case of continuous outcomes.* BMC Medical Research Methodology, 2016. **16**(1): p. 165.
8. Candlish, J., et al., *Appropriate statistical methods for analysing partially nested randomised controlled trials with continuous outcomes: a simulation study.* BMC medical research methodology, 2018. **18**(1): p. 105.
9. Kahan, B.C. and T.P. Morris, *Improper analysis of trials randomised using stratified blocks or minimisation.* Statistics in Medicine, 2012. **31**(4): p. 328-340.
10. Kahan, B.C., et al., *A comparison of methods to adjust for continuous covariates in the analysis of randomised trials.* BMC Medical Research Methodology, 2016. **16**(1): p. 42.
11. Kahan, B.C. and M.O. Harhay, *Many multicenter trials had few events per center, requiring analysis via random-effects models or GEEs.* Journal of clinical epidemiology, 2015. **68**(12): p. 1504-1511.
12. Kahan, B.C. and T.P. Morris, *Analysis of multicentre trials with continuous outcomes: when and how should we account for centre effects?* Stat Med, 2013. **32**(7): p. 1136-49.
13. White, I.R. and S.G. Thompson, *Adjusting for partially missing baseline measurements in randomized trials.* Stat Med, 2005. **24**(7): p. 993-1007.
14. Rubin, D.B., *Inference and missing data.* Biometrika, 1976. **63**(3): p. 581-592.
15. HOCHBERG, Y., *A sharper Bonferroni procedure for multiple tests of significance.* Biometrika, 1988. **75**(4): p. 800-802.
16. Satterthwaite, F.E., *An approximate distribution of estimates of variance components*. Biometrics bulletin, 1946. ***2***(6): p.110-114.
17. Luke, S.G., *Evaluating significance in linear mixed-effects models in R*. Behavior research methods, 2017. **49**(4): p.1494-1502.
18. Bell, M.L., Fairclough, D.L., Fiero, M.H. and Butow, P.N., *Handling missing items in the Hospital Anxiety and Depression Scale (HADS): a simulation study*. BMC research notes, 2016. **9**(1): p.479-488.
19. White, I.R., et al., *Eliciting and using expert opinions about dropout bias in randomized controlled trials.* Clin Trials, 2007. **4**(2): p. 125-39.
20. Norton, S., et al., *The Hospital Anxiety and Depression Scale: a meta confirmatory factor analysis.* J Psychosom Res, 2013. **74**(1): p. 74-81.

8. Appendices

Appendix 1: Data collection

Type of data	Time of data collection	Source and method of data collection	Outcome Measure and type
**Patient participants**			
Demographics	Baseline	Patient participants; supervised self-complete questionnaire	
Clinical:<br>Breathlessness	Baseline	Patient participants; supervised self-complete questionnaire	mMRC Breathlessness scale; categorical
Clinical:<br>Smoking status	Baseline<br>6 months<br>12 months	Patient participants; supervised self-complete questionnaire	Smoking status; categorical
Health status measures	Screening<br>6 months<br>12 months	Patient participants; supervised self-complete questionnaire	HADS-A & HADS-D; continuous
	Baseline<br>6 months<br>12 months	Patient participants; supervised self-complete questionnaire	BDI II, BAI, B-IPQ, SGRQ, heiQ, Time Use Survey (adapted); continuous
PR attendance and completion data	Once following completion of intervention delivery	PR service teams	Attendance and completion data
CBA attendance and completion data	Attendance or failure to deliver recorded at each session during CBA intervention period	Study team/CBA facilitators	Attendance and completion data
**Carer participants**			
Demographics	Baseline	Carer; self-complete questionnaire	
Wellbeing measures	Baseline<br>6 months<br>12 months	Carer; self-complete questionnaires	ZBI, WEMWBS; continuous

Appendix 2: Derived outcomes

Primary outcome measure

HADS questionnaire

There are 14 items, with each item scored from 0-3. The HADS questionnaire has 7 items related to anxiety (HADS-A) and 7 related to depression (HADS-D). Scores are totaled across the anxiety subscale to give a total score for anxiety, and are totaled across the depression subscale to give a total score for depression. Participants can score between 0-21 on each subscale, with higher scores indicating higher anxiety or depression.

Secondary outcomes

BDI-II

There are 21 items, with each item scored from 0-3. The BDI–II is scored by summing the ratings for the 21 items. The minimum score is zero and the maximum total score is 63. Total scores from 0 to 13 reflect minimal depression; 14 to 19 reflect mild depression; 20 to 28 reflect moderate depression; and 29 to 63 reflects severe depression.

BAI

There are 21 items, with each item scored from 0-3. The BAI is scored by summing the ratings for the 21 symptoms. The minimum score is zero and the maximum score is 63. Total scores from 0 to 7 points are considered to reflect a minimal level of anxiety; scores of 8 to 15 indicates mild anxiety; scores of 16 to 25 reflect moderate anxiety; and scores of 26 to 63 indicate severe anxiety.

Smoking status

This is a single question asking the smoking status of the participant. The participant can choose one of three categories for their response: current smoker, ex-smoker, never smoked. For analyses purposes, current smoker forms one category and the categories of ex-smoker and never-smoked are combined together to form the second non-smoker category. Thus, in effect, the categories for smoking status are: smoker and non-smoker.

SGRQ

There are 17 items, each with varying scores. Three component scores are calculated for the SGRQ: Symptoms - this component is concerned with the effect of respiratory symptoms, their frequency and severity. It is calculated from the summed weights for the positive responses to questions 1-8. Activity - concerned with activities that cause or are limited by breathlessness. It is calculated from the summed weights for the positive responses to questions 11 and 15. Impacts - covers a range of aspects concerned with social functioning and psychological disturbances resulting from airways disease. It is calculated from the summed weights for the positive responses to questions 9-10, 12-14, and 16-17. A Total score is also calculated which summarises the impact of the disease on overall health status. It is calculated by summing all positive responses in the questionnaire. The component and total scores are expressed as a percentage of the weight for that component or the total weight, respectively. Scores are expressed as a percentage where 100 represents worst possible health status and 0 indicates best possible health status.

Brief IPQ

There are 8 items, with each item scored from 0-10. Each item of the Brief IPQ assesses one dimension of illness perceptions: The consequences score is simply the response to item 1. The timeline score is the response to item 2. The personal control scores is the response to item 3. The treatment control score is the response to item 4. The identity score is the response to item 5. Illness concern is measured by item 6. The coherence score is the response to item 7. The emotional representation is the response to item 8.

heiQ

There are 5 items on the social integration and support construct of the heiQ questionnaire, each scored from 1-4 (1 = strongly disagree, 2 = disagree, 3 = agree, 4 = strongly agree). The summary score is obtained by summing the scores for all items and dividing by the number of items. The score therefore ranges between 1 and 4, and a higher score indicates better self-management.

Time use survey

There are 12 items, each with a yes/no response about whether the participant has done that activity. There are further sub questions if the answer is yes, which ask the number of times the activity has been done, amount of time, whether it was done with someone, and if so who.

ZBI

There are 22 items, with each item scored from 0-4. Items 1 to 21 are scored as 0 = never and 4 = nearly always, and item 22 is scored as 0 = not at all and 4 = extremely. Scores are summed to give a global score, with a range of 0 to 88.

WEMWBS

There are 14 items, with each item scored from 1-5. A total score is calculated by summing the 14 individual statement scores. The minimum score is 14 and the maximum is 70.

Appendix 3: CONSORT flow diagram

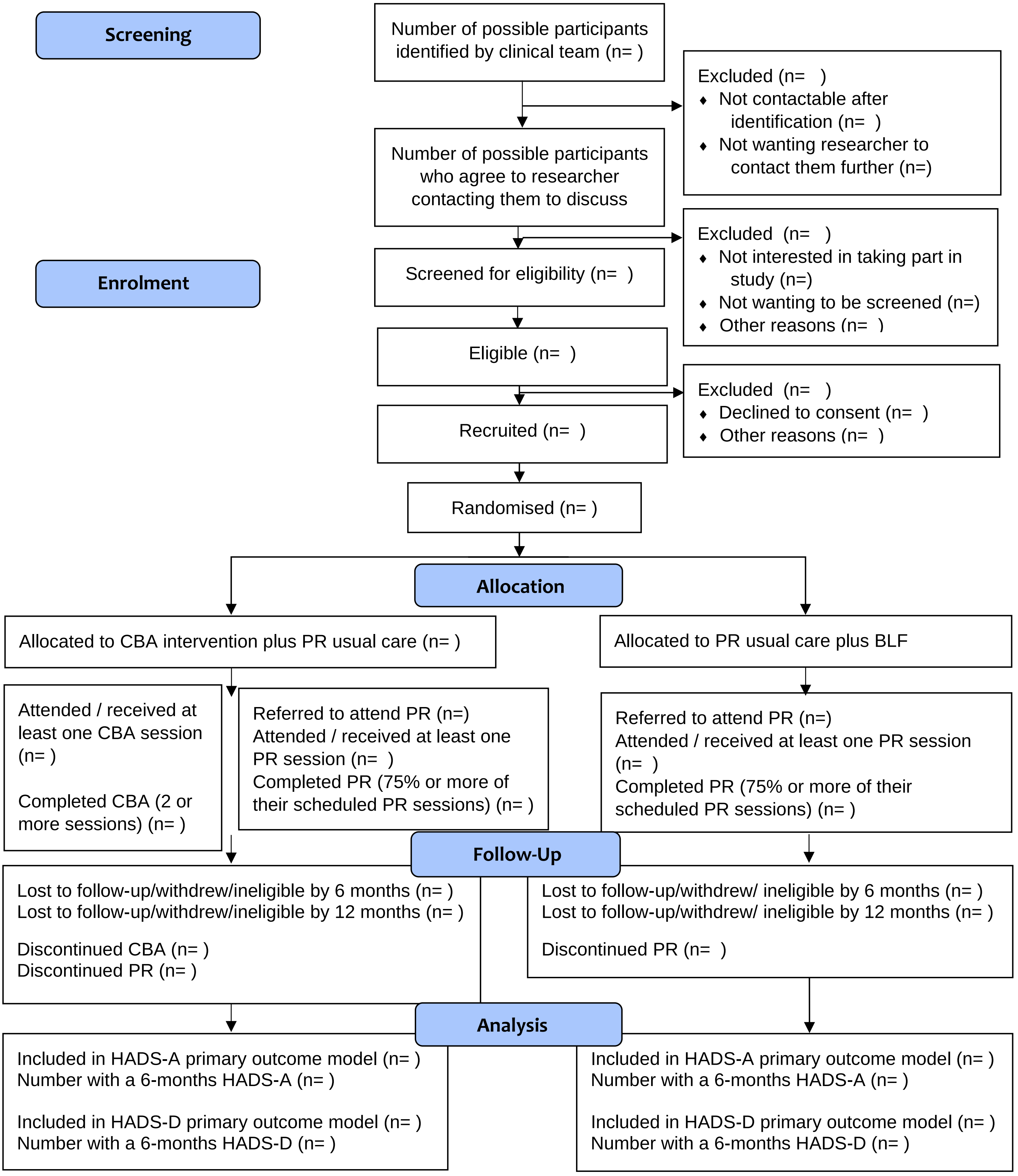

Appendix 4: Dummy tables

Table 1 - Baseline participant demographics and clinical history

	Complete data		Summary measure	
	Intervention no. (%)	Usual care no. (%)	Intervention (n=...)	Usual care (n=...)
Age (years) – mean (SD) median (IQR)				
Male - no. (%)				
Marital status – no. (%)				
Lives alone				
Lives with spouse or partner				
Lives with adult family member				
Lives with spouse or partner and adult family member				
Currently in paid employment/working – no. (%)				
(If yes) Hours per week in paid employment/working – mean (SD) median (IQR)				
Had formal education – no. (%)				
(If yes) Age when completed full-time education (years) – no. (%)				
12 years or under				
13-16				
16-18				
Over 18 years				
On home oxygen – no. (%)				
(If yes) Hours per day on home oxygen – mean (SD) median (IQR)				
(If yes) Type of oxygen equipment used – no. (%)				
Concentrator				
Large cylinder				
Ambulatory				
Age when first diagnosed with COPD – mean (SD) median (IQR)				
Other long-term health problems – no. (%)				
Heart disease				

Diabetes				
Arthritis				
High blood pressure				
Asthma				
Epilepsy				
Other				
Attended a pulmonary rehabilitation course before – no. (%)				
(If yes) Able to complete the course – no. (%)				
Smoking status – no. (%)				
Current smoker				
Ex-smoker				
Never smoked				
(If Current Smoker) Pack years* – mean (SD) median (IQR)				
Current vaper (inc. e-cigarette user) – no. (%)				
Degree of breathlessness (mMRC breathlessness scale) – no. (%)				
Not troubled by breathlessness except on strenuous exercise				
Short of breath when hurrying on the level *or* walking up a slight hill				
Walks slower than most people of the same age on the level because of breathlessness *or* has to stop the breath when walking at own pace on the level				
Stops the breath after walking about 100 yards *or* after a few minutes on the level				
Too breathless to leave the house *or* breathless when dressing or undressing				
Participant had a recent hospitalisation (within last 6 months) for an acute exacerbation of COPD at time of pre-screening†				

Abbreviations: **COPD**: chronic obstructive pulmonary disease
*Pack years is calculated according to the pack years calculator at smokingpackyears.com
†This information was collected part way into the trial so will be missing for some participants

Table 2 - Baseline carer demographics

	Complete data		Summary measure	
	Intervention no. (%)	Usual care no. (%)	Intervention (n=…)	Usual care (n=…)
Age (years) – mean (SD) median (IQR)				
Male - no. (%)				
Relationship to participant – no. (%)				
Son				
Daughter				
Other adult family member				
Spouse or partner				

Table 3 - Baseline participant questionnaires

	Complete data		Summary measure	
	Intervention no. (%)	Usual care no. (%)	Intervention (n=…)	Usual care (n=…)
HADS (Your mood and feelings in the past week)				
HADS-A score – mean (SD)				
HADS-D score – mean (SD)				
BDI-II (Your mood and feelings during the past two weeks including today)				
Sum score – mean (SD)				
BAI (Your worries during the past week including today)				
Sum score – mean (SD)				
SGRQ (Your respiratory health)				
Total score – mean (SD)				
Symptoms score – mean (SD)				
Activity score – mean (SD)				
Impact score – mean (SD)				
B-IPQ (Your views about your illness)				
Consequences score – mean (SD)				
Timeline score – mean (SD)				
Personal control score – mean (SD)				
Treatment control score – mean (SD)				
Identity score – mean (SD)				
Concern score – mean (SD)				
Coherence score – mean (SD)				
Emotional response score – mean (SD)				
heiQ - Social engagement (Your social life)				
Mean score – mean (SD)				
Time Use Survey (Activities in your spare time)				

Time (mins) spent doing activities over last 4 days – mean (SD) median (IQR)				

Abbreviations

HADS: Hospital Anxiety and Depression Scale; **mMRC**: modified Medical Research Council; **BDI-II**: Beck's Depression Inventory-II; **BAI**: Beck's Anxiety Inventory; **SGRQ**: The St Georges Respiratory Questionnaire; **B-IPQ**: The Brief-Illness Perception Questionnaire; **heiQ**: The Health Education Impact Questionnaire

Table 4 - Baseline carer questionnaires

	**Complete data**		**Summary measure**	
	**Intervention no. (%)**	**Usual care no. (%)**	**Intervention (n=…)**	**Usual care (n=…)**
WEMWBS (Your feelings in the last two weeks)				
Sum score – mean (SD)				
ZBI (Your feelings when taking care of another person)				
Sum score – mean (SD)				

Abbreviations

WEMWBS: Warwick-Edinburgh Mental Wellbeing Scale; **ZBI**: Zarit Burden Interview

Table 5 - Minimisation factors within each NHS Trust (separate table per trust)

	**Intervention**	**Usual care**
	**N (%)**	**N (%)**
**Minimisation factors**		
**HADS-Anxiety**		
0 – 7		
8 – 10		
11 – 15		
**HADS-Depression**		
0 – 7		
8 – 10		
11 – 15		
**Modified MRC dyspnoea scale**		
0-2		
3-4		
**Smoking status**		
Smoker		
Non-smoker (ex-smoker/never smoked)		

Table 6 - Completeness of data on questionnaires

	Baseline			6 months			12 months		
	**No. (%) giving data at all**	**No. (%) giving complete data**	**Mean (range) no. of items completed**	**No. (%) giving data at all**	**No. (%) giving complete data**	**Mean (range) no. of items completed**	**No. (%) giving data at all**	**No. (%) giving complete data**	**Mean (range) no. of items completed**
HADS-A (7 items)									
HADS-D (7 items)									
mMRC (1 item)			N/A	N/A			N/A	N/A	
BDI-II (21 items)									
BAI (21 items)									
SGRQ (17 items)									
B-IPQ (8 items)									
heiQ (5 items)									
Time Use Survey (12 items)									
WEMWBS (14 items)									
ZBI (22 items)									

Table 7 - Main results for analysis of primary and secondary continuous outcomes

	Number included in analysis No. (%)		Summary measure Mean (SD)			
	Intervention	Usual Care	Intervention	Usual Care	Treatment effect (95% CI)	p-value
HADS-D at 6 months						
HADS-A at 6 months						
HADS-D at 12 months						
HADS-A at 12 months						
BDI-II at 6 months						
BDI-II at 12 months						
BAI at 6 months						
BAI at 12 months						
SGRQ at 6 months						
Symptoms score						
Activity score						
Impact score						
Total score						
SGRQ at 12 months						
Symptoms score						
Activity score						
Impact score						
Total score						
B-IPQ at 6 months						
Consequences						
Timeline						
Personal Control						
Treatment control						
Identity						
Concern						
Coherence						
Emotional response						
B-IPQ at 12 months						
Consequences						
Timeline						
Personal Control						
Treatment control						
Identity						
Concern						
Coherence						
Emotional response						
heiQ at 6 months						

heiQ at 12 months						
Time Use Survey at 6 months						
Time Use Survey at 12 months						
ZBI at 6 months						
ZBI at 12 months						
WEMWBS at 6 months						
WEMWBS at 12 months						

Table 8 – Smoking status secondary outcome

	**Number included in analysis** No. (%)		**Summary Measure** (Proportion)			
	**Intervention**	**Usual Care**	**Intervention**	**Usual Care**	**Treatment effect (95% CI)**	**p-value**
At 6 months follow-up						
Current smoker						
Non-smoker						
Missing						
At 12 months follow-up						
Current smoker						
Non-smoker						
Missing						

Table 9 – CACE analysis

**CACE estimate**	**Treatment effect (95% CI)**	**P-value**
At 6 months follow-up		
At 12 months follow-up		

Table 10– Results from sensitivity analyses for HADS-A at 6 months

	**Treatment effect (95% CI)**	**P-value**
Main analysis		
Complete case analysis		
Missing items imputed for participants with partially incomplete HADS-A or HADS-D		
Excluding participants with a score <8 on HADS-A subscale		

Time to PR as an additional covariate		
Excluding internal pilot participants		
Randomisation period as an additional covariate		
Pre- *vs* during pandemic (cut-off date 19 Mar 2020) as an additional covariate		
Face-to-face *vs* remote delivery as an additional covariate		

Table 11 – Results from sensitivity analyses for HADS-D at 6 months

	**Treatment effect (95% CI)**	**P-value**
Main analysis		
Complete case analysis		
Missing items imputed for participants with partially incomplete HADS-A or HADS-D		
Excluding participants with a score <8 on HADS-D subscale		
Time to PR as a covariate		
Excluding internal pilot participants		
Randomisation period as an additional covariate		
Pre- *vs* during pandemic (cut-off date 19 Mar 2020) as an additional covariate		
Face-to-face *vs* remote delivery as an additional covariate		

Table 12 - Results of sensitivity analysis for data being MNAR of HADS-A at 6 months

**Assumed mean responses for participants with missing data in usual care group**	**Assumed mean responses for participants with missing data in intervention group**	**Treatment effect (95% CI)**	**Treatment effect adjusted for pandemic (95% CI)**
-10	-15		
	-10		

	-5		
-5	-10		
	-5		
	0		
-1.5	-6.5		
	-1.5		
	3.5		
0	-5		
	0		
	5		
1.5	-3.5		
	1.5		
	6.5		
5	0		
	5		
	10		
10	5		
	10		
	15		

Table 13 - Results of sensitivity analysis for data being MNAR of HADS-D at 6 months

Assumed mean responses for participants with missing data in usual care group	Assumed mean responses for participants with missing data in intervention group	Treatment effect (95% CI)	Treatment effect adjusted for pandemic (95% CI)
-10	-15		
	-10		
	-5		
-5	-10		
	-5		
	0		
-1.5	-6.5		
	-1.5		
	3.5		
0	-5		
	0		
	5		
1.5	-3.5		
	1.5		
	6.5		
5	0		
	5		
	10		

10	5		
	10		
	15		

Table 14 - Comparison of distribution of baseline participant demographics, clinical history and questionnaire characteristics, along with baseline carer demographics under analyses assumptions.

	**Baseline characteristics of participants included in the primary analysis**		**Baseline characteristics of participants with missing primary outcomes at both 6 and 12 months**		**Missing follow-up data due to death**	
	**Intervention**	**Usual Care**	**Intervention**	**Usual Care**	**Intervention**	**Usual Care**
Participant demographics and clinical history						
Age (years) – mean (SD) median (IQR)						
Male - no. (%)						
Marital status – no. (%)						
Lives alone						
Lives with spouse or partner						
Lives with adult family member						
Lives with spouse or partner and adult family member						
Currently in paid employment/working – no. (%)						
(If yes) Hours per week in paid employment/working – mean (SD) median (IQR)						
Had formal education – no. (%)						
(If yes) Age when completed full-time						

education (years) – no. (%)						
12 years or under						
13-16						
16-18						
Over 18 years						
On home oxygen – no. (%)						
(If yes) Hours per day on home oxygen – mean (SD) median (IQR)						
(If yes) Type of oxygen equipment used – no. (%)						
Concentrator						
Large cylinder						
Ambulatory						
Age when first diagnosed with COPD – mean (SD) median (IQR)						
Other long-term health problems – no. (%)						
Heart disease						
Diabetes						
Arthritis						
High blood pressure						
Asthma						
Epilepsy						
Other						
Attended a pulmonary rehabilitation course before – no. (%)						
(If yes) Able to complete the course – no. (%)						
Smoking status – no. (%)						
Current smoker						
Ex-smoker						
Never smoked						

(If Current Smoker) Pack years* – mean (SD) median (IQR)						
Current vaper (inc. e-cigarette user) – no. (%)						
Degree of breathlessness (mMRC breathlessness scale) – no. (%)						
Not troubled by breathlessness except on strenuous exercise						
Short of breath when hurrying on the level *or* walking up a slight hill						
Walks slower than most people of the same age on the level because of breathlessness *or* has to stop the breath when walking at own pace on the level						
Stops the breath after walking about 100 yards *or* after a few minutes on the level						
Too breathless to leave the house *or* breathless when dressing or undressing						
Participant had a recent hospitalisation (within last 6 months) for an acute exacerbation of COPD at time of pre-screening†						
Carer demographics						
Age (years) – mean (SD) median (IQR)						
Male - no. (%)						

Relationship to participant – no. (%)						
Son						
Daughter						
Other adult family member						
Spouse or partner						
Participant questionnaire characteristics						
HADS-A						
HADS-D						
BDI-II						
BAI						
Smoking status						
SGRQ						
Symptoms score						
Activity score						
Impact score						
Total score						
B-IPQ						
Consequences						
Timeline						
Personal Control						
Treatment control						
Identity						
Concern						
Coherence						
Emotional response						
heiQ						
Time Use Survey						

Table 15 - Comparison of distribution of baseline participant demographics, clinical history and questionnaire characteristics, along with baseline carer demographics under different randomisation periods

	**Randomisation Period 1**		**Randomisation Period 2**		**Randomisation Period 3**		**Randomisation Period 4**	
	**Intervention**	**Usual Care**	**Intervention**	**Usual Care**	**Intervention**	**Usual Care**	**Intervention**	**Usual Care**
Participant demographics								

and clinical history								
Age (years) – mean (SD) median (IQR)								
Male - no. (%)								
Marital status – no. (%)								
Lives alone								
Lives with spouse or partner								
Lives with adult family member								
Lives with spouse or partner and adult family member								
Currently in paid employment/ working – no. (%)								
(If yes) Hours per week in paid employment/ working – mean (SD) median (IQR)								
Had formal education – no. (%)								
(If yes) Age when completed full-time education (years) – no. (%)								
12 years or under								
13-16								

16-18								
Over 18 years								
On home oxygen – no. (%)								
(If yes) Hours per day on home oxygen – mean (SD) median (IQR)								
(If yes) Type of oxygen equipment used – no. (%)								
Concentrator								
Large cylinder								
Ambulatory								
Age when first diagnosed with COPD – mean (SD) median (IQR)								
Other long-term health problems – no. (%)								
Heart disease								
Diabetes								
Arthritis								
High blood pressure								
Asthma								
Epilepsy								
Other								
Attended a pulmonary rehabilitation course before – no. (%)								
(If yes) Able to complete the course – no. (%)								

Smoking status – no. (%)								
Current smoker								
Ex-smoker								
Never smoked								
(If Current Smoker) Pack years* – mean (SD) median (IQR)								
Current vaper (inc. e-cigarette user) – no. (%)								
Degree of breathlessness (mMRC breathlessness scale) – no. (%)								
Not troubled by breathlessness except on strenuous exercise								
Short of breath when hurrying on the level *or* walking up a slight hill								
Walks slower than most people of the same age on the level because of breathlessness *or* has to stop the breath when walking								

at own pace on the level								
Stops the breath after walking about 100 yards *or* after a few minutes on the level								
Too breathless to leave the house *or* breathless when dressing or undressing								
Participant had a recent hospitalisation (within last 6 months) for an acute exacerbation of COPD at time of pre-screening†								
Carer demographics								
Age (years) – mean (SD) median (IQR)								
Male - no. (%)								
Relationship to participant – no. (%)								
Son								
Daughter								
Other adult family member								
Spouse or partner								
Participant questionnaire characteristics								
HADS-A								

HADS-D								
BDI-II								
BAI								
Smoking status								
SGRQ								
Symptoms score								
Activity score								
Impact score								
Total score								
B-IPQ								
Consequences								
Timeline								
Personal Control								
Treatment control								
Identity								
Concern								
Coherence								
Emotional response								
heiQ								
Time Use Survey								

Table 16 - Comparison of distribution of baseline participant demographics, clinical history and questionnaire characteristics, along with baseline carer demographics under data splits due to COVID.

	**Split Based on data collection dates**				**Split based on mode of intervention delivery**			
	**Data collected before March 19, 2020**		**Data collected after March 19, 2020**		**Fully face-to-face delivery of intervention**		**Partly remote / face-to-face delivery of intervention**	
	**Intervention**	**Usual Care**	**Intervention**	**Usual Care**	**Intervention**	**Usual Care**	**Intervention**	**Usual Care**
Participant demographics and clinical history								

Age (years) – mean (SD) median (IQR)								
Male - no. (%)								
Marital status – no. (%)								
Lives alone								
Lives with spouse or partner								
Lives with adult family member								
Lives with spouse or partner and adult family member								
Currently in paid employment/ working – no. (%)								
(If yes) Hours per week in paid employment/ working – mean (SD) median (IQR)								
Had formal education – no. (%)								
(If yes) Age when completed full-time education (years) – no. (%)								
12 years or under								
13-16								
16-18								
Over 18 years								

On home oxygen – no. (%)								
(If yes) Hours per day on home oxygen – mean (SD) median (IQR)								
(If yes) Type of oxygen equipment used – no. (%)								
Concentrator								
Large cylinder								
Ambulatory								
Age when first diagnosed with COPD – mean (SD) median (IQR)								
Other long-term health problems – no. (%)								
Heart disease								
Diabetes								
Arthritis								
High blood pressure								
Asthma								
Epilepsy								
Other								
Attended a pulmonary rehabilitation course before – no. (%)								
(If yes) Able to complete the course – no. (%)								
Smoking status – no. (%)								

Current smoker								
Ex-smoker								
Never smoked								
(If Current Smoker) Pack years* – mean (SD) median (IQR)								
Current vaper (inc. e-cigarette user) – no. (%)								
Degree of breathlessness (mMRC breathlessness scale) – no. (%)								
Not troubled by breathlessness except on strenuous exercise								
Short of breath when hurrying on the level *or* walking up a slight hill								
Walks slower than most people of the same age on the level because of breathlessness *or* has to stop the breath when walking at own pace on the level								
Stops the breath after								

walking about 100 yards *or* after a few minutes on the level								
Too breathless to leave the house *or* breathless when dressing or undressing								
Participant had a recent hospitalisation (within last 6 months) for an acute exacerbation of COPD at time of pre-screening†								
Carer demographics								
Age (years) – mean (SD) median (IQR)								
Male - no. (%)								
Relationship to participant – no. (%)								
Son								
Daughter								
Other adult family member								
Spouse or partner								
Participant questionnaire characteristics								
HADS-A								
HADS-D								
BDI-II								
BAI								

Smoking status								
SGRQ								
Symptoms score								
Activity score								
Impact score								
Total score								
B-IPQ								
Consequences								
Timeline								
Personal Control								
Treatment control								
Identity								
Concern								
Coherence								
Emotional response								
heiQ								
Time Use Survey								

Table 97 – CBA and PR attendance and completion

	**CBA intervention**	**PR**
Attendance rate		
Completion rate		

Abbreviations: CBA: Cognitive Behavioural Approach; **PR**: Pulmonary Rehabilitation

Table 108 – Comparison of distribution of time to PR for the two treatment groups

	**Intervention**			**Usual Care**		
	**N**	**Mean**	**S.D.**	**N**	**Mean**	**S.D.**
Time to PR						

Table 19 – Adverse events

	**Intervention N (%)**	**Usual care N (%)**
**Reporting method**<br>Event known to researcher during patient assessment<br>Event know to facilitator during intervention delivery		
**Severity**		

Mild<br>Moderate<br>Severe<br>Life threatening<br>Death		
**Causality**<br>Unrelated<br>Unlikely<br>Possibly<br>Probably<br>Definitely		
**Action taken**<br>No action taken<br>Withdrawal<br>Con Med<br>Non-drug therapy<br>Hospitalisation		
**Outcome**<br>Unresolved<br>Resolving<br>Resolved<br>Fatal<br>Unknown		
**Expectedness**<br>Expected<br>Unexpected		

Table 20 – Serious adverse events

	**Intervention N (%)**	**Usual care N (%)**
**Reporting method**<br>Event known to researcher during patient assessment<br>Event know to facilitator during intervention delivery		
**Severity**<br>Results in death<br>Life threatening<br>Hospitalisation or prolongation of hospitalisation<br>Persistent or significant disability or incapacity<br>Congenital anomaly or birth defect<br>"Other" important medical event		
**Related to one of the procedures in the study**<br>Related<br>Unrelated		
**Expectedness**<br>Expected		

Unexpected		
**Due to the progression of an underlying illness**<br>Yes<br>No		
**Action taken with study treatment and procedures**<br>Continued<br>Reduced<br>Increased<br>Temporary stop<br>Permanent stop		
**Related to the trial conduct**<br>Yes<br>No<br>Not answered		
**PI withdrew the patient from the study**<br>Yes<br>No		
**Outcome**<br>Resolved<br>Resolved with sequelae<br>Improved<br>Persisting<br>Worsened<br>Fatal<br>Unknown		
