# Supplementary material for: Tailored psychological intervention for anxiety or depression in COPD (TANDEM): a randomised controlled trial
Source: Eur Respir J. 2023 Nov 2;62(5):2300432. doi: 10.1183/13993003.00432-2023 (PMC10620475; doi:10.1183/13993003.00432-2023)
Supplement: Supplementary file 3 [file ERJ-00432-2023.Shareable.pdf]

EUROPEAN RESPIRATORY JOURNAL
ORIGINAL RESEARCH ARTICLE
S.J.C. TAYLOR ET AL.

SHAREABLE PDF

Tailored psychological intervention for anxiety or depression in COPD (TANDEM): a randomised controlled trial

Stephanie J.C. Taylor[1,16], Ratna Sohanpal[1,16], Liz Steed[1], Karen Marshall[2], Claire Chan[1], Nahel Yaziji[3], Amy C. Barradell[4], Paulino Font-Gilabert[3], Andrew Healey[3], Richard Hooper[1], Moira J. Kelly[1], Kristie-Marie Mammoliti[5], Stefan Priebe[1], Arvind Rajasekaran[6], C. Michael Roberts[7], Vickie Rowland[8], Sally J. Singh[9], Melanie Smuk[10], Martin Underwood[11,12], Sarah Waseem[13], Patrick White[8], Vari Wileman[14] and Hilary Pinnock[15]

[1]Wolfson Institute of Population Health, Queen Mary University of London, London, UK. [2]Chest Clinic, RVI Hospital, Newcastle upon Tyne NHS Foundation Trust, Newcastle upon Tyne, UK. [3]Health Service and Population Research Department, Institute of Psychiatry, Psychology and Neuroscience, King's College London, London, UK. [4]NIHR Leicester Biomedical Research Centre – Respiratory, Glenfield Hospital, University Hospitals of Leicester NHS Trust, Leicester, UK. [5]WHO Collaborating Centre on Global Women's Health, Institute of Metabolism and Systems Research, University of Birmingham, Birmingham, UK. [6]Department of Respiratory Medicine, Sandwell and West Birmingham Hospitals NHS Trust, Birmingham, UK. [7]Centre for Digital Transformation of Health, University of Melbourne, Melbourne, Australia. [8]Department of Population Health, School of Life Course and Population Sciences, King's College London, London, UK. [9]Department of Respiratory Sciences, Department of Health Sciences, University of Leicester, Leicester, UK. [10]Blizard Institute, Queen Mary University of London, London, UK. [11]Warwick Clinical Trials Unit, University of Warwick, Coventry, UK. [12]University Hospitals of Coventry and Warwickshire, Coventry, UK. [13]Women's Health Division, University College Hospital, London, UK. [14]Health Psychology, School of Mental Health and Psychological Sciences, Institute of Psychiatry, Psychology and Neuroscience, King's College London, London, UK. [15]Allergy and Respiratory Research Group, Usher Institute, The University of Edinburgh, Edinburgh, UK. [16]Joint first authors.

Corresponding author: Stephanie J.C. Taylor (s.j.c.taylor@qmul.ac.uk)

Check for updates

Shareable abstract (@ERSpublications)
The TANDEM trial reports no benefit from a cognitive behavioural approach intervention focused particularly on breathlessness in people with advanced COPD and anxiety/depression. More research is needed for these patients with high levels of unmet need. https://bit.ly/3QDCB8v

Cite this article as: Taylor SJC, Sohanpal R, Steed L, *et al*. Tailored psychological intervention for anxiety or depression in COPD (TANDEM): a randomised controlled trial. *Eur Respir J* 2023; 62: 2300432 [DOI: 10.1183/13993003.00432-2023].

This extracted version can be shared freely online.

Copyright ©The authors 2023.

This version is distributed under the terms of the Creative Commons Attribution Licence 4.0.

This article has an editorial commentary: https://doi.org/10.1183/13993003.01538-2023

Received: 10 March 2023
Accepted: 8 Aug 2023

Abstract

Background The TANDEM multicentre, pragmatic, randomised controlled trial evaluated whether a tailored psychological intervention based on a cognitive behavioural approach for people with COPD and symptoms of anxiety and/or depression improved anxiety or depression compared with usual care (control).

Methods People with COPD and moderate to very severe airways obstruction and Hospital Anxiety and Depression Scale subscale scores indicating mild to moderate anxiety (HADS-A) and/or depression (HADS-D) were randomised 1.25:1 (242 intervention and 181 control). Respiratory health professionals delivered the intervention face-to-face over 6–8 weeks. Co-primary outcomes were HADS-A and HADS-D measured 6 months post-randomisation. Secondary outcomes at 6 and 12 months included: HADS-A and HADS-D (12 months), Beck Depression Inventory II, Beck Anxiety Inventory, St George's Respiratory Questionnaire, social engagement, the EuroQol instrument five-level version (EQ-5D-5L), smoking status, completion of pulmonary rehabilitation, and health and social care resource use.

Results The intervention did not improve anxiety (HADS-A mean difference −0.60, 95% CI −1.40–0.21) or depression (HADS-D mean difference −0.66, 95% CI −1.39–0.07) at 6 months. The intervention did not improve any secondary outcomes at either time-point, nor did it influence completion of pulmonary rehabilitation or healthcare resource use. Deaths in the intervention arm (13/242; 5%) exceeded those in the control arm (3/181; 2%), but none were associated with the intervention. Health economic analysis found the intervention highly unlikely to be cost-effective.

Conclusion This trial has shown, beyond reasonable doubt, that this cognitive behavioural intervention delivered by trained and supervised respiratory health professionals does not improve psychological comorbidity in people with advanced COPD and depression or anxiety.
